# *In silico* design and evaluation of a candidate multiepitope *Salmonella* vaccine construct targeting broad-spectrum protection in poultry

**DOI:** 10.1186/s12864-026-13056-4

**Published:** 2026-06-25

**Authors:** David J. Bradshaw, Melissa S. Monson, Bradley L. Bearson, Shawn M. D. Bearson

**Affiliations:** 1 USA Department of Agriculture, Agricultural Research Service, National Animal Disease Center, Ames, IA United States; 2 Oak Ridge Institute for Science and Education, Agricultural Research Service Participation Program, Oak Ridge, TN USA; 3 USA Department of Agriculture, Agricultural Research Service, National Laboratory for Agriculture and the Environment, Ames, IA United States

**Keywords:** *Salmonella enterica* subspecies *enterica*, Reverse vaccinology, Poultry, *In silico* vaccine design, *Salmonella* outbreak isolates, Multiepitope vaccine

## Abstract

**Background:**

*Salmonella enterica* subspecies *enterica* is an important source of human foodborne illness, frequently via contaminated food animal products. Vaccination is a promisingly effective intervention to lower *Salmonella* loads in food animals, thus reducing food chain transmission. Currently-available commercial vaccines have limited cross protection across *Salmonella* serovars (> 2,600), indicating a need for novel vaccines with broad efficacy. In this study, an *in silico* reverse vaccinology (RV) pipeline comprehensively screened the proteome of *Salmonella enterica* serovar Typhimurium strain UK-1 for conserved proteins from poultry-associated, human-relevant *Salmonella* serovars and identified epitopes with predicted broad-spectrum protection for the design of a multiepitope vaccine construct (MEVC).

**Results:**

The UK-1 proteome, excluding lipopolysaccharide-, flagellin-, and plasmid-associated proteins, was screened for proteins with pertinent properties including homology to five poultry-associated, human-relevant serovars (Enteritidis, Hadar, Infantis, Kentucky, and Uganda), representing *Salmonella* serogroups B-E. The resulting 101 proteins were evaluated for cytotoxic and helper T lymphocyte epitopes with strong binding to chicken-like human major histocompatibility complex alleles, high antigenicity, and 100% identity to ≥ 99% of the NCBI proteomes of the selected serovars (*n* = 90,800). Of the resulting 93 epitopes, 28 epitopes derived from 24 proteins were incorporated in an MEVC with epitope-type-associated linkers and a *Salmonella* flagellin adjuvant. *In silico* tools predicted immunological functions of the MEVC including TLR1/TLR2 binding, induction of classical cellular and humoral immune responses, and sequence homology (i.e. potential cross-protection) to a *Salmonella* outbreak dataset.

**Conclusions:**

A modified RV pipeline using a whole proteome approach targeting human-relevant, poultry-associated serovars identified previously recognized proteins with immunogenic and/or immunoprotective properties (suggestive of *in vivo* protein expression and immune stimulation), as well as eight novel proteins for vaccine target exploration. Our RV pipeline was further corroborated by 25 of the 28 MEVC epitopes demonstrating 100% sequence identity to > 90% of a PulseNet dataset of 135 outbreak-associated *Salmonella* serovars from various food sources. Although efficacy validation of the proposed MEVC requires *in vivo* or *in vitro* evaluation, the current study illustrates the utility of pairing target organisms with relevant datasets to *in silico* screen, assemble, and assess a multiepitope vaccine construct designed for predicted broad-spectrum protection.

**Supplementary Information:**

The online version contains supplementary material available at https://doi.org/10.1186/s12864-026-13056-4.

## Background

*Salmonella enterica* is a ubiquitous, Gram-negative, pathogenic bacterium classified into > 2,600 serovars based on variability in the flagellar H antigens and cell envelope lipopolysaccharide (LPS) O antigen [1]. Serovars are summarized into O-antigen-based serogroups, with five serogroups (A, B, C, D, and E) causing 99% of infections in humans and warm-blooded animals [2]. *Salmonella* is divided into typhoidal or non-typhoidal *Salmonella* (NTS) based on the clinical presentation of human-associated infections: an invasive, life-threatening, systemic disease (typhoid fever) or an occasionally invasive but usually self-limiting diarrheal gastroenteritis (salmonellosis), respectively. NTS is estimated to cause 150 million cases of salmonellosis associated with 60,000 deaths worldwide each year [3]. In the United States, the Centers for Disease Control and Prevention (CDC) estimates NTS causes 1.35 million illnesses, 26,500 hospitalizations, and 420 deaths annually, accounting for 23.6% of foodborne illnesses and a projected $17.1 billion in associated costs (USDA Economic Research Service) [4, 5].

The most recent annual foodborne illness source attribution publication by the U.S. Interagency Food Safety Analytics Collaboration estimates that chicken consumption has the highest source attribution percentage (19.7%) to *Salmonella* outbreaks followed by pork (4th, 11.9%), beef (6th, 6.9%), and turkey (7th, 5.2%), thereby reflecting the importance of interventions to reduce *Salmonella* colonization in food animals [6, 7]. Unrecognized *Salmonella* colonization, transmission, and contamination occur in food animal production and processing environments because *Salmonella* frequently establishes a subclinical carrier state in food animals, resulting in the foodborne pathogen stealthily entering the food chain [8]. For example, unnoticed *Salmonella* fecal shedding can lead to transmission within the flock/herd as well as contamination of agricultural products consumed raw (fruits, vegetables, spices) when fertilized with *Salmonella*-contaminated soil amendments of animal origin (manure, litter, etc.) or due to improperly treated irrigation water [9]. Furthermore, *Salmonella* is a potential reservoir and vehicle for antibiotic resistance and metal tolerance genes (when present) to neighboring bacteria via mechanisms such as conjugation and phage-mediated gene transfer, which may be induced by antibiotic exposure [10]. Previous studies have also suggested that low levels of antibiotics can lead to upregulation of virulence genes in *Salmonella* facilitating its survival in the host [11]. Thus, non-antibiotic intervention strategies to control *Salmonella* in food animals, especially poultry (24.9% total source attribution), are warranted to reduce the human pathogen in our food supply and minimize the consequences of antibiotic resistance.

Vaccination is a promising intervention tool to lower *Salmonella* loads in food animals, thereby reducing food chain transmission and contamination. A major limitation of current *Salmonella* vaccines is the lack of cross-protection against the > 2,600 serovars due to the wide variability of specific immunodominant cell surface antigens [8]. Multivalent vaccines expressing antigens with conserved homology across the major *Salmonella* serogroups could provide immunological cross protection to the vaccinated host [12]. In the current study, reverse vaccinology (RV) was used to design a multiepitope vaccine construct with potential broad-spectrum protection to reduce *Salmonella* in poultry. Using *in silico* tools, RV employs subtractive proteomics and immunoinformatic approaches to design and evaluate a multiepitope vaccine candidate containing statistically-selected, antigenic epitopes for strategic activation of a wide array of host cellular and humoral immune responses [13]. Along with the potential to design for broad protection, the efficient and cost-effective vaccine development of RV offers a rapid response to emerging serovars in food animals or in human outbreaks [12, 14].

To enhance the biological relevance and robustness of the epitope selection process, the current study incorporated modifications to the conventional four phases of vaccine design (Subtractive Proteomics, Epitope Identification and Filtering, Construct Design, and Assessment of Construct Properties) to identify and optimize physical, structural, and immunological characteristics of the proposed vaccine construct by including: (1) interrogating a complete *Salmonella* proteome but excluding protein descriptions that contain LPS or flagella to remove these immunovariable and immunodominant antigens, (2) incorporating chicken-like human alleles into the epitope selection process, (3) robustly testing epitopes for sequence conservation to relevant *Salmonella* serovars, (4) utilizing dual-purpose linear B lymphocyte epitopes, (5) ensuring selection of a docking model that incorporated biological relevance, and (6) adding a fifth Phase (Conservation of Construct Epitopes) focused on assessing epitope sequence homology to outbreak-associated *Salmonella* isolates. Using the proteome of the well-studied, pathogenic *Salmonella enterica* subspecies *enterica* serovar Typhimurium (*S*. Typhimurium) strain UK-1 (serogroup B) [15, 16], focus was placed on identifying homologous epitopes across human-relevant and poultry-associated serovars to *in silico* design and assess (using a PulseNet outbreak dataset) [17, 18] a multiepitope vaccine candidate with potential broad protection against *Salmonella* in poultry.

## Materials and methods

### Data management and statistical analysis

The background and technical information regarding tools used in this study is provided in the Supplementary Materials and Methods section (Additional File 1). Additionally, throughout the pipeline, RStudio (2023.06.0; R v. 4.4.1) was used for data processing and statistical analysis. The RStudio environment was loaded with the following main libraries: tidyverse (v. 2.0.0), Biostrings (v. 2.72.1), ape (v. 5.8), scales (v. 1.3.0), Peptides (v. 2.4.6), seqinr (v. 4.2–36), plyr (v. 1.8.9), lubridate (v. 1.9.3), foreach (v. 1.5.2), parallel (v. 4.4.1), and doParallel (v. 1.0.17) [19–29] Command line scripts, R scripts, and R Studio session information including additional supportive libraries used and their versions are available on GitHub (https://github.com/USDA-FSEPRU/rev_vacc_salm_mevc.git).

### Phase I – Subtractive proteomics

#### Objective

**Fig. 1:**
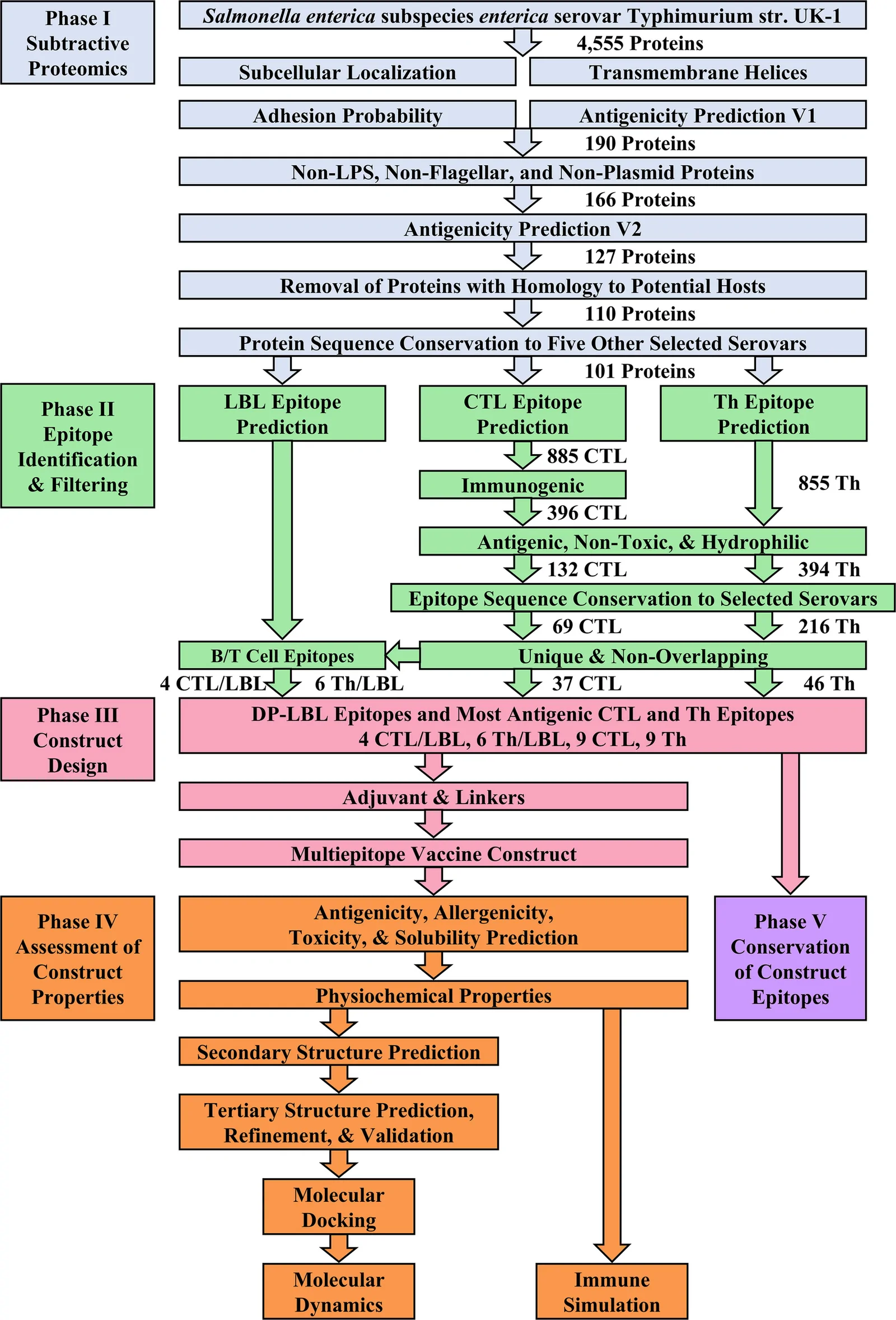
Flowchart of reverse vaccinology multiepitope vaccine construct design. CTL = cytotoxic T lymphocyte, Th = helper T lymphocyte, DP-LBL = dual purpose linear B lymphocyte

Chromosome-encoded proteins from a representative NTS (*S.* Typhimurium) genome were filtered for outer membrane/extracellular/periplasmic proteins with high adhesion probability, high antigenicity score, exclusion of protein descriptions for LPS, flagella, or plasmids, little to no homology to potential host proteins, and sequence conservation across proteins from human-relevant and poultry-associated serovars (Fig. 1 – blue section). The tools used for Phase I and all subsequent Phases are summarized in Supplementary Table 1 (Additional File 2).

### Proteome retrieval

The complete proteome (4,555 proteins) of *S.* Typhimurium strain UK-1 (GenBank Assembly GCA_000213635.1) was used as the input query into the RV pipeline [15].

### Vaxign2

Vaxign2 is an online server used in RV to assess proteins as potential vaccine targets [30, 31]. Vaxign2 contains both the Vaxign Machine Learning (ML) method (in-house antigenicity prediction tool) and various third-party tools to evaluate protein properties [31, 32]. *S.* Typhimurium str. UK-1 proteins (faa file) were input into Vaxign2 under the “Bacterium” and “Gram negative bacterium” Pathogen Organism Type with the following analysis options: Subcellular Localization, Transmembrane Helix, Adhesion Probability, and Vaxign-ML Analysis. Filtering parameters were based on developer recommendations and RV literature [13, 31–33].

A threshold of 90.0 for the Vaxign-ML antigenicity score was used, and localization was determined with PSORTb (v3.0) [34]. This workflow focused on surface exposed and secreted protein types (Outer Membrane, Extracellular, and Periplasmic) due to a higher probability of their interaction with the host immune system [35]. Adhesion probability was determined by SPAAN using a threshold of ≥ 0.51; adhesions are considered good vaccine targets due to their conserved nature and importance in pathogenesis [36–38]. The number of trans-membrane helices was determined by TMHMM (v2.0) [39], and the Vaxign2 server recommended threshold of 0–1 transmembrane helices was used because proteins with multiple transmembrane helices are difficult to clone and express [37, 39, 40].

### Protein identity

Many of the proteins in the GenBank assembly of *S.* Typhimurium str. UK-1 were not annotated with protein names. Thus, in order to capture the potential biological roles of antigenic proteins, additional annotations were added to the UK-1 proteins through alignment similarity (percent identity ≥ 98%) to other *Salmonella enterica* subsp. *enterica* proteins (last accessed 07/03/23) (43,234,599 proteins; taxid:59,201) via BLASTp (v. 2.14.1) with default parameters (Accession Numbers Table 1 – Additional File 3) and by adding the RefSeq Assembly annotations for the same assembly (GCF_000213635.1) [41–43]. UK-1 proteins that contained flagella, LPS, or plasmid in their protein descriptions (GenBank, RefSeq, or similar *Salmonella* proteins (SSP)) were manually excluded from further analysis because of the transient nature of plasmids and the immunovariability of *Salmonella* LPS and flagellar proteins (> 2,600 *Salmonella* serovars are differentiated by variability in their LPS and flagellar antigens). This leads to the exclusion of highly variable serovar-defining antigens in favor of conserved antigens.

### VaxiJen2

In order to ensure that proteins were considered antigenic by multiple methods, further screening of the outer membrane, extracellular and periplasmic proteins for protective antigen prediction was performed using the VaxiJen (v. 2.0) server at a threshold of ≥ 0.5 [30, 33, 44–46]. While VaxiJen (v. 2.0) and Vaxign-ML both employ machine learning, they were trained on different datasets, thus offering different interpretations on antigenicity.

### Removal of proteins with homology to potential hosts

Vaccine targets should be non-homologous to potential host proteins to avoid autoimmune reactions. Although the focus of this study was to design a multiepitope vaccine candidate for use in poultry, it could be utilized or evaluated in additional *Salmonella* hosts if its administration is effective in poultry. Thus, all antigenic proteins from *S.* Typhimurium str. UK-1 were subjected to BLASTp against the major species where the vaccine could potentially be used or tested (last accessed 07/03/23): *Gallus gallus* (chicken) (159,892 proteins; taxid:9031), *Meleagris gallopavo* (turkey) (30,663 proteins; taxid:10,090), *Sus scrofa* (pig) (98,748 proteins; taxid:9823), *Bos taurus* (cow) (145,913 proteins; taxid:9913), *Mus musculus* (mouse) (362,575 proteins; taxid:10,090), and *Homo sapiens* (human) (1,907,751 proteins; taxid:9606). BLASTp was used to remotely query the non-redundant protein database against each target species; max target sequences were set to 10 and all other parameters set to default. UK-1 proteins with an Expect value (E-value) less than 1e-4 were considered to have homology to a host protein and removed from the study [13]. Host protein accession numbers from the top ten alignments to UK-1 proteins generated during analysis are found in Accession Numbers Table 2 (Additional File 3).

### Protein sequence conservation with select serovars

For RV to predict a *Salmonella* vaccine with potential broad-spectrum protection for poultry, proteins selected from *S.* Typhimurium str. UK-1 need to be highly conserved across human-relevant and poultry-associated *Salmonella* serovars. Five serovars representing four serogroups were chosen based upon their prevalence in the most relevant food animal species (*Gallus gallus, Meleagris gallopavo*) and their importance in human infections: Serogroup D *S.* Enteritidis (4,241,000 proteins; taxid:149,539), Serogroup C1 *S*. Infantis (963,345 proteins; taxid:595), Serogroup C2 *S*. Hadar (326,970 proteins; taxid:149,385), Serogroup C2 *S*. Kentucky (1,123,476 proteins; taxid:192,955), and Serogroup E *S*. Uganda (94,853 proteins; taxid:487,004). BLASTp was used to remotely query the non-redundant protein database for each target serovar; max target sequences were set to 10 and all other parameters set to default. Serogroup B *S*. Typhimurium UK-1 proteins with a percent identity ≥ 98%, per previous literature, across each of the 5 target serovars were considered homologous and selected for Phase II [41]. *Salmonella* serovar protein accession numbers from the top ten alignments to UK-1 proteins generated during analysis are found in Accession Numbers Table 3 (Additional File 3).

### Phase II – Epitope identification and filtering

#### Objective

Selected UK-1 proteins from Phase I were assessed for 9-mer cytotoxic T lymphocyte (CTL), 15-mer helper T lymphocyte (Th), and linear B lymphocyte (LBL) epitope predictions. The predicted epitopes were evaluated for immunogenicity, antigenicity, non-toxicity, and hydrophilicity as well as high amino acid sequence conservation with human-relevant and poultry-associated *Salmonella* serovars (Fig. 1 – green).

### Selection of major histocompatibility complex alleles

Because the resulting vaccine candidate will be initially tested in poultry and chicken alleles are not available in the prediction tools, the human major histocompatibility complex (MHC) class I (MHCI) and class II (MHCII) alleles with closest homology to chicken MHCI (BF) and MHCII (BL) molecules should be employed to predict epitopes. A recent study determined the most appropriate alleles to extrapolate to chickens by aligning all human MHCI and MHCII alleles in NetMHCpan and NetMHCIIpan against the most frequent haplotypes of chicken MHC alleles from the assessed meat-type birds [47]. They determined that the best performing human leukocyte antigen (HLA) B isotype MHCI alleles were HLA-B*40:06, HLA-B*41:04, and HLA-B*41:03 and the best performing HLA DRB isotype MHCII alleles were HLA-DRB1*13:10, HLA-DRB1*13:66, HLA-DRB1*14:45, and HLA-DRB1*14:82.

### CTL and Th epitope prediction

Using the MHC alleles noted above, the NetMHCpan (v.4.1) and NetMHCIIpan (v.4.0) servers were used to identify 9-mer CTL and 15-mer Th epitopes, respectively, within the selected UK-1 proteins using the following thresholds: strong binder at ≤ 0.5% Rank and weak binder at 0.5–2% Rank, which are more stringent than the default thresholds (1% and 5%) for NetMHCIIpan. All other settings were kept at the default.

### Immunogenicity and antigenicity

The Immune Epitope Database (IEDB) Class I Immunogenicity web server was used with default parameters to determine which CTL epitopes were considered immunogenic, as indicated by a positive score [48]. Antigenicity was determined for both CTL and Th epitopes with VaxiJen (v. 2.0) with a threshold of ≥ 0.5.

### Toxicity and hydrophobicity

The ToxinPred server was used with default parameters in the dipeptide support vector machines (SVM)-based method to determine whether the remaining CTL and Th epitopes were toxic or non-toxic [49]. ToxinPred also provides information on hydrophobicity, hydropathicity, hydrophilicity, charge, and molecular weight by default. Epitopes were selected if their grand average of hydropathy (GRAVY) score was negative [50], indicating that the epitope was hydrophilic and thus more likely to be surface exposed and recognized by the immune system [13].

### Epitope sequence conservation to select serovars

BLASTp was used to determine which epitopes had 100% sequence identity to a minimum percentage (assessed at 100%, 99%, 98%, 97%, 96%, 95%, and 90%) of the publicly available NCBI proteomes (NCBI Assembly database last accessed on 07/03/23) from each of the following human-relevant and poultry-associated serovars: *S.* Enteritidis (37,000 proteomes, taxid:149,539), *S.* Infantis (14,052 proteomes, taxid:595), *S.* Hadar (1,304 proteomes, taxid:149,385), *S.* Kentucky (11,000 proteomes, taxid:192,955), *S.* Typhimurium (25,983 proteomes, taxid:90,371), and *S.* Uganda (961 proteomes, taxid:487,004) (Accession Numbers Table 4 – Additional File 3). Proteomes were downloaded with the NCBI Entrez Direct tool (v.16.2) [51]. Default BLASTp parameters were used except for max target sequences set to 5 and E-value limit to 10,000 to accommodate the short length of query epitopes leading to high E-values. The filter for epitopes that have 100% identity to ≥ 99% of proteomes within each serovar for all six serovars was chosen for the pipeline.

### Unique and non-overlapping epitopes

Duplicated epitopes predicted for multiple MHC alleles were identified and combined by protein sequences into unique epitopes. If partially overlapping sequences between unique epitopes were identified within a protein, only the epitope with the highest VaxiJen antigenicity was selected. This selection protocol was chosen to prioritize antigenicity (with magnitudes that allowed selection between epitopes) because most of the other epitope metrics were pass/fail.

### Dual purpose linear B cell epitope prediction and filtering

B lymphocyte epitopes exist in two types: linear/continuous (LBL) and conformational/discontinuous (CBL). Studies have shown that 90% of B-cell epitopes are confirmational, but most have sequential residue stretches [13, 52]. BepiPred (v3.0) predicted LBL residues from the Phase I UK-1 proteins with default parameters using sequential smoothing (linear epitope prediction mode). Only LBL epitopes that were also predicted as CTL or Th epitopes were included to design dual-purpose LBL (DP-LBL) epitopes to effectively utilize construct space. The minimum length of a LBL epitope is 8 residues, and up to 25 residues [53], thus a 9-mer CTL or 15-mer Th epitope was also considered an LBL epitope if it had ≥ 8 continuous BepiPred-identified LBL residues. Despite being used in many RV studies [54, 55], CBL prediction was not included in this study as the protein structure of the multiepitope vaccine construct (MEVC) would not recapitulate the conformation of the full-length bacterial proteins and therefore would not likely contribute to protection induced by the vaccine.

### Phase III – Construct design

#### Objective

Epitopes with the greatest antigenicity were combined with linkers and an adjuvant to develop a multiepitope vaccine construct (MEVC) (Fig. 1 – pink).

### Multiepitope vaccine construct epitope region design

From the filtered antigenic, non-toxic, hydrophilic, unique, non-overlapping CTL and Th epitopes with conserved homology to all six tested serovars, the nine CTL and nine Th epitopes with the highest antigenicity were selected. All DP-LBL epitopes were used to construct the epitope region. The sequence for a proprietary *Salmonella* flagellar adjuvant (495 aa) was included to assist with activation of the immune system.

### Linkers

Construct linkers separating the components of the MEVC were chosen based on consistent usage across RV studies where the linkers enhanced immunogenic processes and construct stability [56–58]. AAY was used as the CTL linker because it enhances proteolytic cleavage and release of the epitopes for binding to the transporter associated with antigen processing (TAP) and subsequent MHCI loading [59]. GPGPG was used to link the CTL and Th regions and between Th epitopes because these amino acids are unfavorable to cross-epitope (junctional) binding by mammalian MHCII, which conserves relevant epitopes for presentation to helper T-cells [59]. KK was used between the Th and DP-LBL regions as well as between DP-LBL epitopes because it is a target of lysosomal proteases during processing of epitopes for presentation via MHCII for induction of antibody production [60]. EAAAK was used as the linker between the epitope region and the proprietary adjuvant and after the final DP-LBL epitope at the C terminal of the construct to reduce interaction with other protein regions [61].

### Phase IV – Assessment of construct properties

#### Objective

The MEVC was assessed *in silico* for potential physical and chemical properties, antigenicity, allergenicity, solubility, toxicity, and secondary structure prediction (Fig. 1 – orange). Tertiary structure was predicted, refined, validated, and evaluated for docking to toll-like receptor 1/2 (TLR1/2); the Construct-TLR 1/2 complex was then tested for stability. The MEVC was further utilized in an immune simulation.

### Antigenicity, allergenicity, solubility, and toxicity

Antigenicity was predicted with VaxiJen2 using the threshold of 0.5. Allergenicity of the MEVC protein sequence was assessed using the AllergenFP server [62], and solubility of the construct was evaluated with SolPro from the SCRATCH Protein Predictor server [63, 64]. A higher solubility suggests better purification during downstream processing, an important factor in post-production vaccine studies [65]. The ToxinPred2 server predicted the toxicity of the MEVC protein sequence; ToxinPred2 is a protein-focused update of ToxinPred which assesses only peptides and small proteins [66].

### Physical and chemical characteristics

The Expasy-ProtParam web tool predicted the physical and chemical characteristics of the MEVC based on its protein sequence including: molecular weight, theoretical pI, amino acid composition, atomic composition, instability index, aliphatic index, and GRAVY score [67]. Although most of these parameters are descriptive, the instability (threshold > 40 is unstable) and aliphatic indexes assess the instability and thermostability of the construct, respectively [68, 69].

### Secondary structure prediction

PSIPRED (v. 4.0), which is part of the PSIPRED Workbench Server, predicted secondary structure for the MEVC from its protein sequence as well as the percentages of substructures such as alpha-helices, beta-strands, and/or coils [70, 71].

### Tertiary structure prediction and refinement

The Phyre (v. 2.0) server used the MEVC protein sequence to predict tertiary structure in intensive mode [72]. Visualization of this model and subsequent models was performed with ChimeraX (v. 1.9) [73]. The GalaxyRefine (v. 1.0) server refined the original Phyre tertiary structure [74]. GalaxyRefine reported metrics to compare the original model to 5 new models. Global Distance Test-High Accuracy (GDT-HA) measures the protein backbone similarity to the initial model, with scores closer to 1 indicating alternative models closer to the initial model [74, 75]. Root mean square deviation (RMSD) values closer to 0 indicate alternative models closer to the initial model [75]. Smaller MolProbity values indicate that the model has accurate geometric predictions of amino acids and bonding angles that are comparable to known crystal protein structures and balances amino acids and angles in locations that are favorable for that amino acid but minimizes clashing with other amino acids in the structure [76]. The model with the best combination (summed rank) of GDT-HA (highest, i.e. closer to original model), RMSD (lowest, i.e. closer to the original model), and MolProbity (lowest, i.e. balancing clashing and favored scores) scores was chosen for further analysis. If a tie in summed rank occurred, then the MolProbity score was chosen as the tiebreaker metric because it is a compilation of a number of metrics and covers more variables than the RMST or GDT-HA scores.

### Tertiary structure validation

The SAVES (v. 6.0) server was used to run validation checks using ERRAT, VERIFY3D, and PROCHECK on pre-refined and refined structures. ERRAT is based on relative frequencies of noncovalent contacts between atoms while VERIFY3D is based on the compatibility of a 3D model with its sequence, respectively [77–79]. ERRAT and VERIFY3D scores greater than 50% and 80%, respectively, are indications of acceptable models. PROCHECK generates a Ramachandran plot of residue φ–ψ torsion angles for the model shaded based upon the frequency of the residue angles within the database: most favored, allowed, generously allowed, and disallowed. The distribution of the query residues across these shaded regions allows for evaluation of the quality of the structure in comparison to high-resolution protein structures [80, 81]. ProSA-web generates a z-score to measure the deviation in total energy [82]. Structure Z-scores closer to the center of the random conformation energy distribution are considered better quality models.

### Molecular docking

Due to the inclusion of epitopes from bacterial lipoproteins in the construct, MEVC binding to the TLR1/2 heterodimer was predicted by molecular docking and dynamics. Poultry possess two TLR2 proteins that form heterodimers with either of two TLR1 proteins to recognize di- or tri-acylated lipopeptides from bacteria [83]. TLR1/2 has been used for molecular docking of *Salmonella*-associated or poultry-associated constructs in other studies [14, 56, 84, 85]. The reference crystal structure for human TLR1-TLR2 ectodomain heterodimer bound to a tri-acylated lipopeptide (2Z7X) was downloaded from the RCSB Protein Data Bank to test for docking with the MEVC following manual curation to remove any ligands, water, small structures or molecules not associated with the main ectodomain [86]. Structural analyses have shown that dimerization of TLR1 and TLR2 occurs upon attachment of the ligand to the binding pocket on the outer convex surface on each contributing TLR, corresponding to the leucine rich regions (LRR) 9, 10, 11, and 12; thus the heterodimer (Chains A and B) was used for molecular docking [86, 87].

The ClusPro server (v 2.0) “Balanced” option was used to predict molecular docking with the refined MEVC structure as the ligand and the curated TLR1/2 heterodimer as the receptor [88–91]. ClusPro suggests selection of the representative docking model from the cluster with the most members (i.e. models) for further analysis. To add biological relevance to selection of the most appropriate ClusPro docking model for the MEVC, interacting areas in ClusPro docking models between the MEVC and the curated TLRs were compared to interacting areas between the reference crystal structure for the TLR1/2 heterodimer and its reference ligand (tri-acylated lipopeptide). Due to the expectation that there will be binding residue differences associated with different hosts (TLRs) or ligands (bacterial species or serovar), docking models were assessed for docking to the general interacting area identified in the reference crystal structure instead of screening for specific interacting residues [83]. Models were evaluated in order of decreasing ClusPro cluster members through TLR-MEVC residue interaction analysis and visualization with PDBsum and through tertiary structure generation with ChimeraX [92]. The first docking model visually confirmed to (1) have PDBsum-identified MEVC interactions with both TLR1 and TLR2 and (2) visually bind to the biologically relevant region from the reference crystal structure was selected for further evaluation. This ensured that TLR docking of the MEVC was analogous to the biologically relevant reference.

### Molecular dynamics

Molecular dynamics was performed on the selected TLR1/2-MEVC complex to assess stability, binding, and dynamics of the docked complexes using GROMACS (v2023.3) [93, 94]. Parameters and procedure for GROMACS were adapted from previous studies [95–97]. Physiological conditions were simulated at 1 bar for physiological pressure and 0.15 M salt concentration based on human simulations, as poultry values are not defined in the literature, and at 41.5 °C (314.65 K) based on a normal body temperature in chickens between 41 and 42 °C [98–103]. First, topology was generated with an Optimized Potential for Liquid Simulation All Atom LMP2 (OPLS-AA/L) force field and the extended simple point charge model (SPC/E) as the water model [13, 104, 105]. During the system preparation phases, the model was placed in a virtual simulation box with a distance from the edge set to at least 1.0 nm and SOL as the solvent molecule, and neutralized with Na^+^ or Cl^−^ ions to a physiological concentration (0.15) [101–103]. The second phase of molecular dynamics included several simulations: energy minimization, constant number of particles, volume, and temperature (NVT) equilibration, constant number of particles, pressure, and temperature (NPT) equilibration, and finally a production run. In the energy minimization simulation, potential energy of the system was minimized to less than 1000 kJ/mol/nm to ensure a reasonable starting structure in terms of geometry and solvent orientation. The 100 ps NVT (V-rescale thermostat) and NPT (Parrinello-Rahman barostat) simulations were used to equilibrate the system to a constant temperature (314.65 K) and pressure (1 bar), respectively [98–100]. The 50 ns production run simulation was conducted with the position restraints released on the heavy atoms, a V-rescale thermostat, a Parrinello-Rahman barostat, a constant temperature of 314.65 K, a constant pressure of 1 bar, and a time step of 2 fs [99, 103, 106]. RMSD values (based on the protein complex’s carbon backbone groups) were calculated as an indication of complex stability for the NVT, NPT, and production run simulations. Calculations of root mean square fluctuation (RMSF) values (C-alpha groups) as an indication of side chain flexibility, and the radius of gyration values (whole protein groups) as an indication of structure compactness were performed only for the production run.

### Immune simulation

The C-ImmSim server was used to run an *in silico* vaccination simulation to predict the immune response to the vaccine construct [107–110]. This simulation was run with one MHC molecule for each of the three types of host HLAs available on the server: HLA-A (MHC class I), HLA-B (MHC class I), and HLA-DRB (MHC class II). Since none of the human alleles for HLA-A were identified as chicken-like (40), the default HLA-A*01:01 from C-ImmSim was used, as well as the following randomly selected pairs of alleles from the chicken-like human HLA-B and HLA-DRB alleles used in epitope prediction: HLA-B*41:03 and HLA-DRB1*13:66; HLA-B*41:04 and HLA-DRB1*14:45; and HLA-B*40:06 and HLA-DRB1*13:10. The final chicken-like HLA-DRB1*14:82 allele was not available in C-ImmSim. The simulation was run for 252-time steps of 8 h each (84 days) and two vaccine construct (no LPS) injections were given at time steps 1 (Day 0) and 63 (Day 21); all other parameters were set to default.

### Phase V – Conservation of construct epitopes

#### Objective

Construct epitopes were tested for sequence conservation with PulseNet outbreak-associated isolates (suggestive of potential broad-spectrum protection).

Whole genome sequencing metadata associated with *Salmonella* outbreak strains (*n* = 33,764) was received on 03/01/24 from the CDC PulseNet Network [17, 18]. The *Salmonella* serovar identification/designation was originally determined by PulseNet with SeqSero2. Keywords were used to query the PulseNet isolate metadata (source site, source type, and type details) to summarize isolates based on isolation sources of interest (egg, chicken, turkey, human, cattle/beef, and swine/pork). The same keywords were used to query the outbreak source metadata to summarize isolates based on outbreak sources of interest, which were the same as isolation sources, with the addition of poultry for outbreak sources that did not specify between egg, chicken, or turkey sources. Isolates with metadata that did not include one of these keywords were summarized as “Other” for both isolation and outbreak sources.

SRR ids (Accession Numbers Table 5 – Additional File 3) were used to download raw sequences from the Sequence Read Archive (SRA); ten isolates were removed due to invalid SRR ids. Fastp (v. 0.23.3) was used for quality trimming, SKESA (v. 2.4.0) was used for assembly (*n* = 33,754), and CheckM (v. 1.2.2) was used to assess assembly completeness and contamination [111–113]. Bakta (v. 1.8.2) was used to annotate and generate proteomes for assemblies with a completeness ≥ 95% and a contamination ≤ 5% (*n* = 33,672; BLAST queries); resulting proteomes were aligned against the 28 MEVC epitopes using BLASTp with default parameters except for max target sequences set to 5 and E-value limit to 10,000. Results were filtered for alignments with complete homology, meaning they had 100% identity (percentage of amino acid matches in the alignment) and 100% subject coverage (percentage of the epitope that aligned with each PulseNet proteome). The number of MEVC epitopes with complete homology alignments was assessed at 99% and 90% of the PulseNet proteomes overall, on a per serovar basis and on the basis of isolation or outbreak source.

## Results

### Phase I – Subtractive proteomics analysis

To initiate the RV pipeline, the 4,555 proteins of the *S.* Typhimurium UK-1 proteome were filtered to identify proteins with properties that predict a high probability of contributing to immunological responses across *Salmonella* serovars and serogroups. Selection for high adhesion probabilities, high predicted antigenicity, 0–1 transmembrane helices, and outer membrane, extracellular, or periplasmic localizations resulted in 190 proteins (Fig. 1, Phase I—blue) (Vaxign2). Proteins with flagellar, LPS, or plasmid protein descriptions (*n* = 24) were removed due to high variability between *Salmonella* serovars. VaxiJen was used as a second antigenicity filter, removing 40 proteins, while homology to potential hosts (*Gallus gallus*, *Meleagris gallopavo*, *Sus scrofa*, *Bos taurus*, *Mus musculus*, and *Homo sapiens*) and a lack of protein conservation across human-relevant and poultry-associated serovars (*S.* Enteritidis, *S.* Hadar, *S.* Infantis, *S.* Kentucky, and *S.* Uganda) removed 17 and 9 proteins, respectively. Of the remaining 101 proteins, 79 were annotated with gene names based on GenBank, SSP, or RefSeq annotations, while 22 lacked an annotated gene name but did have protein descriptions. Localization analysis predicted 24 proteins to be extracellular, 50 to be outer membrane-associated, and 27 to be periplasmic (Supplementary Table 2 – Additional File 2).

### Phase II – Epitope identification and filtering

Epitopes from the resulting 101 UK-1 proteins of Phase I were predicted and filtered for properties involved in immune response induction (Fig. 1, Phase II – green). T cell epitope prediction resulted in 885 CTL (NetMHCpan) and 855 Th (NetMHCIIpan) epitopes with strong binding to one or more of the three MHCI or four MHCII chicken-like human alleles, respectively. These epitopes represented 91 and 72 proteins, respectively, for a total of 92 unique proteins. CTL epitopes were further filtered for immunogenicity (IEDB), resulting in 396 epitopes from 76 proteins. Additional filtering for antigenicity (VaxiJen), non-toxicity (ToxinPred), and hydrophobicity (GRAVY) yielded 132 CTL and 394 Th epitopes, from 51 and 57 proteins, respectively, to screen for sequence conservation with six *Salmonella* serovars representing four serogroups (B-E) with clinical relevance in human infections and a pronounced prevalence in poultry.

Requiring epitopes to have BLAST alignments with 100% identity to all proteomes within the NCBI database for six selected *Salmonella* serovars (*S*. Enteritidis, *S*. Typhimurium, *S*. Infantis, *S*. Kentucky, *S*. Hadar, and *S*. Uganda) eliminated all epitopes for further analysis, so 100% identity to proteome coverages of 90–99% were assessed (Table 1). To balance the confidence in achieving cross-reactivity while having sufficient epitopes for construct design, epitopes with 100% sequence identity to 99% of the proteomes within each of the six serovars were chosen as the filter, resulting in 69 CTL and 216 Th epitopes derived from 53 proteins.

**Table 1:** Number of epitopes with 100% identity to varying percentages of proteomes for each serovar

	Number of Epitopes with 100% Identity to													
	100% of Serovar Proteomes		99% of Serovar Proteomes		98% of Serovar Proteomes		97% of Serovar Proteomes		96% of Serovar Proteomes		95% of Serovar Proteomes		90% of Serovar Proteomes	
*Salmonella* Serovar	FALSE	TRUE	**FALSE**	**TRUE**	FALSE	TRUE	FALSE	TRUE	FALSE	TRUE	FALSE	TRUE	FALSE	TRUE
Enteritidis (*n* = 37,000)	526	0	**99**	**427**	83	443	81	445	81	445	81	445	81	445
Hadar (*n* = 1,704)	292	234	**54**	**472**	49	477	39	487	39	487	39	487	36	490
Infantis (*n* = 14,052)	526	0	**109**	**417**	83	443	67	459	67	459	67	459	63	463
Kentucky (*n* = 11,100)	522	4	**95**	**431**	85	441	65	461	63	463	63	463	63	463
Typhimurium (*n* = 25,983)	521	5	**107**	**419**	55	471	41	485	27	499	26	500	18	508
Uganda (*n* = 961)	293	233	**107**	**419**	107	419	105	421	82	444	82	444	82	444
Within Each Serovar for All Six Serovars	526	0	**241**	**285**	196	330	163	363	136	390	135	391	132	394

Bolded text indicates the threshold chosen for further analysis

Approximately 40% of these epitopes were duplicated sequences identified for more than one of the chicken-like MHC alleles used in Phase II. Therefore, epitopes were combined based on protein sequence to find unique epitopes (CTL = 42, Th = 126); overlapping epitopes within the same protein were further filtered to select the epitope with the highest antigenicity (CTL = 41, Th = 52). Resulting epitopes were also screened for the presence of LBL residues; 9-mer CTL and 15-mer HTL epitopes with greater than 8 continuous LBL residues were also considered LBL epitopes, resulting in 10 DP-LBL epitopes (CTL/LBL = 4, Th/LBL = 6) each from a unique protein (Supplementary Table 3 – Additional File 2). Of the remaining unique and highly antigenic epitopes not classified as DP-LBL, 37 were CTL and 46 were Th epitopes (Fig. 1). All together, these unique, non-overlapping DP-LBL, CTL, and Th epitopes represented 53 proteins.

### Phase III – Construct design

**Fig. 2:**
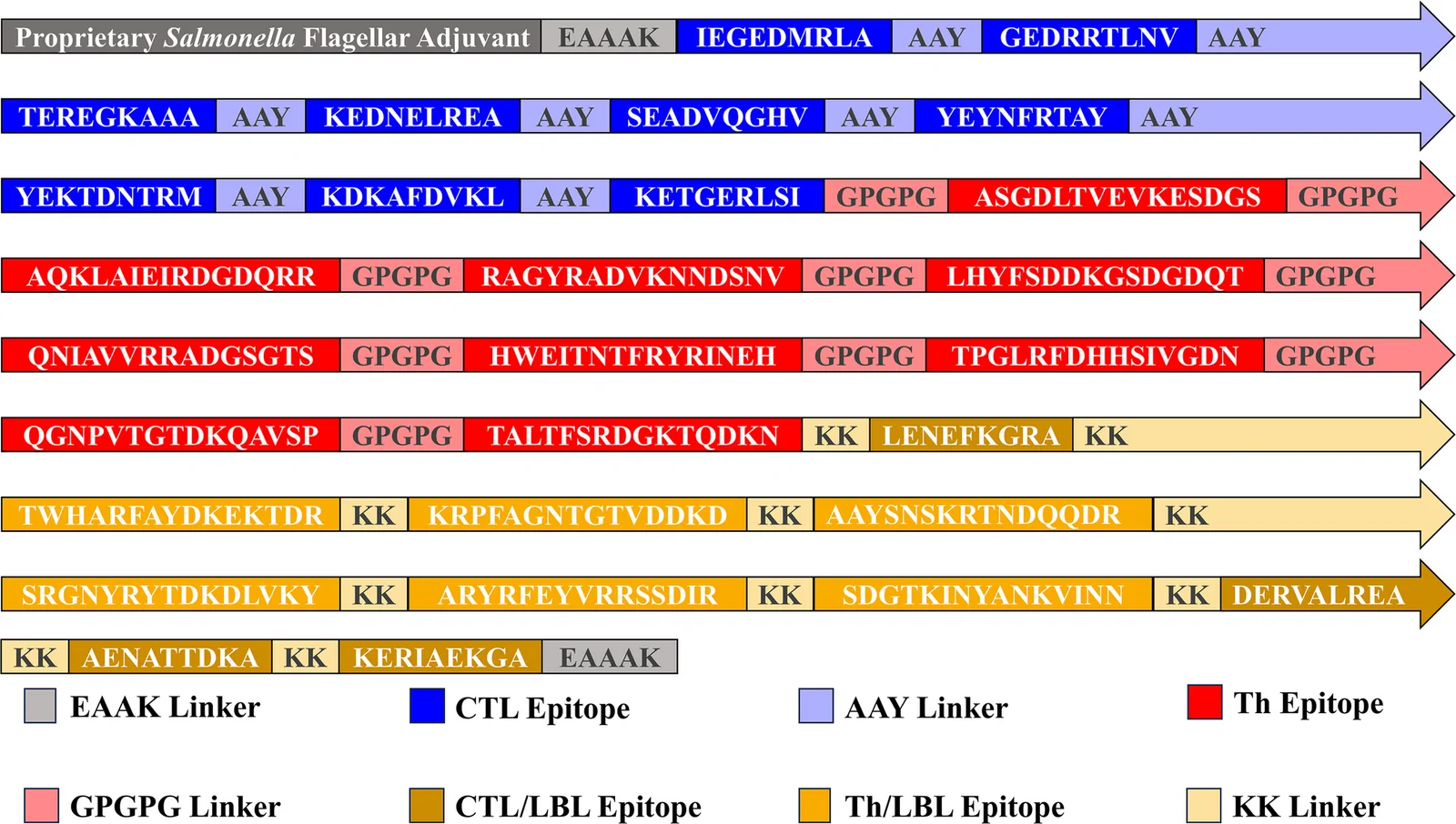
Schematic representation of final multiepitope vaccine construct design. Color represents epitope and linker types. CTL = cytotoxic T lymphocyte, Th = helper T lymphocyte, LBL = linear B lymphocyte

All ten DP-LBL epitopes along with the most antigenic non-LBL CTL (*n* = 9) and Th (*n* = 9) epitopes were chosen to include in the proposed MEVC (Fig. 1, Phase III – pink; Table 2; Fig. 2). The total length of the MEVC was 936 amino acids with the multiepitope region accounting for 441 amino acids. The 28 epitopes selected for the MEVC represented 24 *Salmonella* proteins of which five were not annotated with a specific gene/protein name. Two proteins were predicted to be extracellular, six to be periplasmic, and sixteen to be outer membrane (Table 2). None of the construct epitopes had 100% identity to 100% of the *S.* Enteritidis, *S.* Infantis, or *S.* Kentucky proteomes, but 2, 19, and 21 epitopes had 100% identity to 100% of the *S.* Typhimurium, *S.* Hadar, and *S.* Uganda proteomes, respectively (Supplementary Table 4 – Additional File 2).

**Table 2:** Cytotoxic T lymphocyte (CTL), helper T lymphocyte (Th), and dual-purpose T/B lymphocyte (DP-LBL) epitope properties

GenBank Accession	Epitope Type	MHC HLA Allele	Start Position*	Peptide	Epitope VaxiJen Score	Protein Name(s)^	GenBank Protein Description	Protein Localization	Immunological Properties^#^
AEF05957.1	Th	DRB1*13:10 DRB1*13:66	337	ASGDLTVEVKESDGS	1.69	BcfC, FimD	fimbrial usher	Outer Membrane	RV
AEF06160.1	CTL/LBL	B*40:06	46	LENEFKGRA	1.18	Skp, OmpH	periplasmic chaperone	Periplasmic	AT IG IP
AEF06480.1	Th	DRB1*13:10 DRB1*13:66	109	AQKLAIEIRDGDQRR	1.63	None	putative fimbrial protein	Extracellular	
AEF06499.1	Th	DRB1*13:10 DRB1*13:66	598	TALTFSRDGKTQDKN	1.29	None	outer membrane esterase	Outer Membrane	
AEF06514.1	CTL	B*40:06 B*41:03 B*41:04	620	KETGERLSI	1.46	FepA	outer membrane receptor FepA	Outer Membrane	IG RV
	Th	DRB1*14:82	449	TPGLRFDHHSIVGDN	1.33				
	Th	DRB1*14:82	666	QGNPVTGTDKQAVSP	1.30				
AEF06556.1	Th/LBL	DRB1*13:10 DRB1*13:66	60	TWHARFAYDKEKTDR	1.00	PagP	palmitoyl transferase	Outer Membrane	AT
AEF06700.1	Th/LBL	DRB1*13:10	381	KRPFAGNTGTVDDKD	0.83	None	putative pectinesterase	Outer Membrane	
AEF06873.1	Th/LBL	DRB1*13:10	210	AAYSNSKRTNDQQDR	1.40	OmpF	outer membrane protein F precursor	Outer Membrane	IG IP RV AT
AEF07457.1	CTL	B*40:06	56	IEGEDMRLA	2.47	PqqU, YncD	putative outer membrane receptor	Outer Membrane	AT
AEF07866.1	Th/LBL	DRB1*13:10 DRB1*13:66	334	SRGNYRYTDKDLVKY	0.71	OmpC, OmpS1	putative porin	Outer Membrane	IG IP RV AT
AEF08184.1	Th	DRB1*13:10	41	LHYFSDDKGSDGDQT	1.50	OmpC	outer membrane porin protein C	Outer Membrane	IG IP RV AT
AEF08270.1	CTL	B*40:06	223	KEDNELREA	1.68	HisJ	histidine transport protein	Periplasmic	AT
AEF08636.1	CTL	B*41:03 B*41:04	327	YEKTDNTRM	1.47	FepA	outer membrane receptor FepA	Outer Membrane	IG RV
AEF08671.1	CTL	B*40:06 B*41:03 B*41:04	300	SEADVQGHV	1.59	ProX	glycine betaine transporter periplasmic subunit	Periplasmic	
AEF08880.1	CTL	B*41:03 B*41:04	793	GEDRRTLNV	1.89	StdB	putative outer membrane usher protein	Outer Membrane	RV
AEF09108.1	CTL	B*40:06	163	TEREGKAAA	1.76	OsmY, YraP	hypothetical protein STMUK_3251	Periplasmic	AT
AEF09689.1	Th	DRB1*14:45	153	QNIAVVRRADGSGTS	1.41	PstS	phosphate ABC transporter periplasmic substrate-binding protein PstS	Periplasmic	IG IP
AEF09827.1	Th	DRB1*14:82	184	HWEITNTFRYRINEH	1.33	OmpL/YshA	outer membrane porin L	Outer Membrane	IG IP
AEF09863.1	CTL	B*40:06 B*41:03 B*41:04	184	YEYNFRTAY	1.50	None	putative outer membrane protein	Outer Membrane	
	Th/LBL	DRB1*13:10 DRB1*13:66	127	ARYRFEYVRRSSDIR	1.03				
AEF10024.1	CTL	B*41:04	103	KDKAFDVKL	1.47	None	putative outer membrane lipoprotein	Outer Membrane	
AEF10030.1	Th/LBL	DRB1*14:45	286	SDGTKINYANKVINN	0.68	LamB	maltoporin	Outer Membrane	IG IP
AEF10041.1	Th	DRB1*13:10 DRB1*13:66	385	RAGYRADVKNNDSNV	1.54	TraF	conjugative transfer protein	Outer Membrane	
	CTL/LBL	B*40:06	188	DERVALREA	0.83				
AEF10350.1	CTL/LBL	B*40:06	28	AENATTDKA	1.33	OsmY	periplasmic protein	Periplasmic	
AEF10384.1	CTL/LBL	B*40:06	56	KERIAEKGA	0.67	SthA	putative fimbrial chaperone	Extracellular	IG RV

*MHC* Major histocompatibility complex, *HLA* Human leukocyte antigen, *CTL* Cytotoxic T lymphocyte, *Th* Helper T lymphocyte, *LBL* Linear B lymphocyte

^*^Start position refers to the amino acid position within the antigenic protein where the epitope begins

^Protein Names from one or more of three sources: RefSeq, GenBank, or Similar *Salmonella* Proteins (≥ 98% sequence identity via BLASTp)

^#^Immunological Properties: RV indicates the protein or epitope was identified as a vaccine construct candidate in a reverse vaccinology (RV) study, AT (attenuation target) indicates that modification or deletion of the protein attenuated bacterium infection, IG indicates the protein was immunogenic (IG) in a host, and IP indicates a protein was immunoprotective (IP) for a host following a subsequent bacterial challenge

NetMHCpan and NetMHCIIpan predicted that all the chicken-like HLA MHCI (*n* = 3) and MHCII (*n* = 4) alleles bind to at least two of the MEVC epitopes (Table 2). Five or six CTL epitopes were projected to bind each MHCI allele, while all four CTL/LBL epitopes were predicted to bind to HLA-B*40:06. The MHCII alleles were more variable in Th and Th/LBL epitope binding, with the most predictions for HLA-DRB1*13:10 (five Th and five Th/LBL) and the least for HLA-DRB1*14:45 (one each Th and Th/LBL). HLA-DRB1*14:82 was the only MHCII allele not projected to bind at least one Th/LBL epitope. Additional information regarding the MEVC epitopes including NetMHCpan/NetMHCIIpan and ToxinPred scores as well as additional annotation details (SSP annotations, RefSeq annotation, and gene ontology) are in Supplementary Table 5 (Additional File 2).

Proteins must contain suitable CTL, Th, and LBL epitopes to establish robust cellular and humoral immune responses and induce persistent immunological memory. While the RV pipeline was designed to logically choose epitopes based upon desirable traits, a search was performed to determine if the proteins represented in the MEVC have been previously identified by other immunological studies, thereby providing additional support for their usage in the MEVC. Of the 24 proteins represented by the 28 epitopes within the MEVC, 16 MEVC proteins were previously recognized in RV publications (Table 2) [13, 41, 84, 114–120]. Investigations from prior research in chickens [41, 121, 122], mice [116, 123–127], and zebrafish [128] described immunogenic (IG) properties for ten proteins in the MEVC. Seven of the immunogenic proteins, one (Skp) of which was also identified as a virulence attenuation target (AT) [129], were immunoprotective (IP) against *Salmonella* and other bacterial species in various hosts including chickens (*Pasteurella multicida* strain X-73, *S.* Enteritidis, and *S.* Gallinarum biovar Pullorum strain R51) [41, 121, 122], mice (*S*. Typhi, *S*. Typhimurium, and *S.* Paratyphi A CMCC 50973) [124–127], and zebrafish (*Vibrio* spp.) [128]. Seven other MEVC proteins (PapP, YncD, HisJ, YraP, OmpC, OmpF) were identified as potential virulence attenuation targets in *Salmonella* or other bacteria [130–136]. Additional information regarding the previously identified immunological properties of MEVC proteins is found in Supplementary Table 6 (Additional File 2).

### Phase IV – Assessment of construct properties

#### Physiochemical and biological properties

An array of analytical software tools was used to predict if the proposed MEVC had desirable physiochemical and biological properties (Fig. 1, Phase IV – orange). The MEVC was predicted to be antigenic, non-allergenic, non-toxic, soluble, alkaline, stable, hydrophilic, and relatively thermostable (Table 3).

**Table 3:** Multi epitope vaccine construct physiochemical and biological properties

Metric	Tool	Score	Value or Result
Antigenicity	VaxiJen2^#^	VaxiJen2 Score	1.0348
Allergenicity	AllergenFP	Prediction	Probable Non-Allergen
		Tanimoto Similarity Index	0.88
		UniProtKB Accession Number	Q9NXV6
Toxicity	ToxinPred2	ML Score	0.43
		MERCI Score	0
		Blast Score	−0.5
		Hybrid Score	−0.07
		Prediction	Non-Toxin
Solubility	SolPro	Score	0.772325
		Prediction	Soluble
Size	ExPASy-ProtParam	Amino Acid Number	936
		Molecular Weight (kDa)	99.75176
Charge		Positively Charged Residues	127
		Negatively Charged Residues	118
Alkalinity/Acidity		Theoretical pI	8.88
Stability*		Instability Index	23.06
Thermostability		Aliphatic Index	66.11
Hydrophilicity^		GRAVY Score	−0.772

^#^The VaxiJen2 threshold for a protein to be considered antigenic is ≥ 0.5

^*^The instability index threshold for a protein to be considered to be unstable ≥ 40

^A protein is considered hydrophilic with negative Grand average of hydropathy (GRAVY) scores

#### Secondary structure prediction

PSIPRED was used to acquire basic metadata on the distribution of secondary structure features within the MEVC protein sequence. The 2D structure of the MEVC was predicted to have 45.19% of the amino acids involved in forming coils (423 aa), 39.96% forming helices (374 aa), and 14.85% forming β-strands (139 aa) (Supplementary Fig. 1 – Additional File 4).

#### Tertiary structure prediction, refinement, and validation

**Fig. 3:**
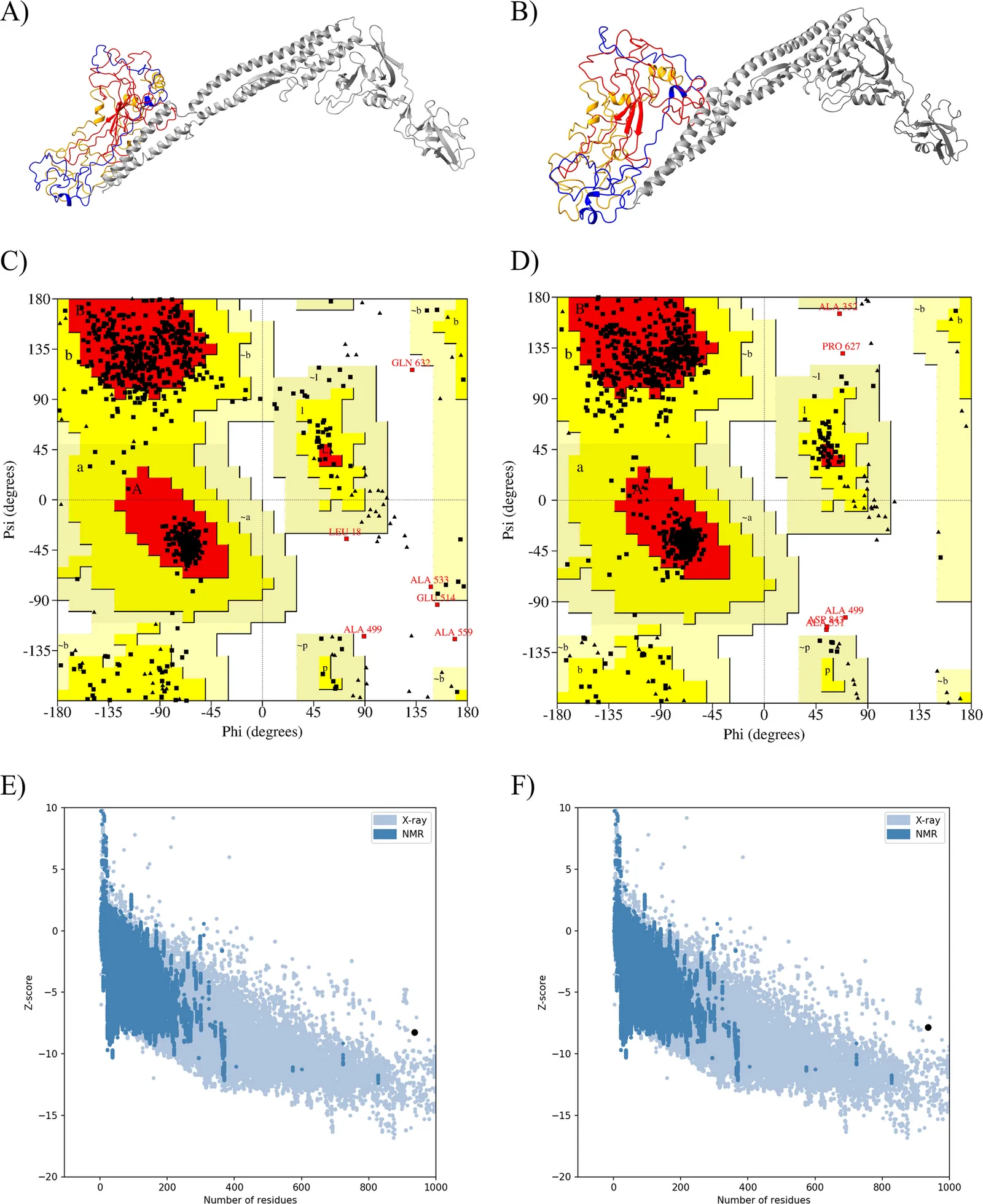
Tertiary structure prediction, refinement, and validation of multiepitope vaccine construct (MEVC). **A** Phyre2 predicted 3D structure of MEVC and (**B**) GalaxyRefine refined 3D structure of MEVC visualized by ChimeraX software with gray representing the adjuvant, blue the cytotoxic T cell epitope region, red the helper T cell epitope region, and orange the dual-purpose linear B cell epitope region. Ramachandran plots of (**C**) original and (**D**) refined MEVCs generated by PROCHECK on the PDBsum server illustrating the distribution of the sterochemical properties of MEVC amino acid residues across the most favored or core (red), additional allowed (yellow), generously allowed (light yellow), and disallowed (white) regions in comparison to database of high-resolution protein structures. Black triangles indicate glycine residues. Black squares are amino acids within the most favored, additionally allowed, or generously allowed regions. Red squares are amino acids within the disallowed region and are labeled with the amino acid type and position. Different regions are labeled as described in Morris et al. (1992): **A** core alpha, **a** allowed alpha, ~ **a** generous alpha, **B** core beta, **b** allowed beta, ~ **b** generous beta, **L** core left-handed alpha, **l I** allowed left-handed alpha, ~ **l)** generous left-handed alpha, **p)** allowed epsilon, and ~ **p** generous epsilon. ProSA server z-score plot with (**E**) original and (**F**) refined MEVCs represented by a black dot illustrating how the z-score (representing the MEVC’s total energy) deviates from an energy distribution derived from random X-ray (light blue) or NMR (dark blue) derived conformations. A value closer to the center of this distribution indicates a better-quality model that more closely matches known structures. Note: The z-score (black dot) for the original structure (**E**: −7.84) is slightly higher in the graph than the z-score for the refined structure (**F**: −8.26) indicating the refined model is a better-quality model

Tertiary structure prediction of the MEVC involved an initial structure prediction followed by refinement that adjusts the relative positions of residues and side chains to suggest alternative structures to evaluate for improvements. The tertiary structure of the MEVC was originally modeled by Phyre2 (Fig. 3A) and refined by GalaxyRefine (Fig. 3B). From the five refined models, GalaxyRefine Model 4 tied for the lowest summed rank (6) of GDT-HA, RMSD, and MolProbity scores with GalaxyRefine Model 2 but had a lower MolProbity score than Model 2; thus, GalaxyRefine Model 4 was chosen to conduct further analysis and validation (Table 4). Importantly, all scores for GalaxyRefine Model 4 (except outlier rotamers) improved on the original Phyre2 model scores. Furthermore, Model 4 was the refined model with the best combination of minimizing changes from the original prediction (GDT-HA and RMSD) while also minimizing clashing between residues and optimizing the favored rotamer percentages.

**Table 4:** GalaxyRefine values for predicted tertiary structure models

Model	GDT-HA	GDT-HA Rank	RMSD	RMSD Rank	MolProbity	MolProbity Rank	Clash score	Poor rotamers	Rama favored	Ranks Sum
Initial	1	NA	0	NA	2.814	NA	52	0.1	86.8	NA
Model 1	0.9589	3	0.396	3	1.95	1	7.3	0.8	89.9	7
Model 2	0.9658	1	0.384	1	1.99	4	7.9	0.7	89.6	6
Model 3	0.9583	4	0.396	3	1.99	4	8	0.4	89.7	11
**Model 4**	**0.9607**	**2**	**0.393**	**2**	**1.968**	**2**	**7.7**	**0.6**	**90**	**6**
Model 5	0.9578	5	0.397	5	1.988	3	8.2	0.7	90.3	13

*GDT-HA* Global Distance Test—High Accuracy, *RMSD* Root mean square deviation

Initial model was predicted by Phyre2 while Models 1–5 were predicted by GalaxyRefine

Bolded text indicates the model chosen for further analysis

1–5 in GDT-HA Rank, RMSD Rank, and MolProbity Rank columns indicate the favorable ranking score for each measurement with 1 indicating the most favorable score

Various tertiary structure validation tools were used to ensure that the predicted refined model was similar to other known structures in the tool’s databases and demonstrated improved metrics compared to the original model. The PROCHECK Ramachandran plot statistics improved in the refined model, visualized by a higher percentage of residues in the most favored regions and a lower percentage of residues in the disallowed regions (Fig. 3C & D) (Table 5). Both the initial and refined models had ERRAT and VERIFY3D values greater than the minimum quality threshold for the two measurements, and the ProSA Z-scores were within the visual normal range of other models with the same number of residues, indicating good overall model quality (Fig. 3E & F). The refined model was employed in downstream analysis because it improved upon the validation scores of the original model, and it was considered a quality model based on appropriate thresholds.

**Table 5:** Tertiary structure validation metrics

Model	PDB sum—PROCHECK				SAVES		ProSA-Web
	Most favored regions	Additional allowed regions	Generously allowed regions	Disallowed regions	ERRAT	VERIFY3D	
Original Phyre Model	80.10%	14.60%	4.60%	0.70%	51.92%	81.84%	−7.84
GalaxyRefine Model 4	83.80%	13.70%	2.10%	0.50%	72.43%	82.08%	−8.26

PROCHECK compares the query’s stereochemical properties to high-resolution proteins structures from the Protein Data Bank

ERRAT evaluates the relative frequencies of noncovalent (non-bonded) contacts between atoms; threshold = 50%

VERIFY3D measures the compatibility of a 3D model with its sequence; threshold = 80%

ProSA-web evaluates the deviation in total energy of a structure from an energy distribution of known structures. The z-score for the structure should be visually within the range of values for known structures

#### Molecular docking

**Fig. 4:**
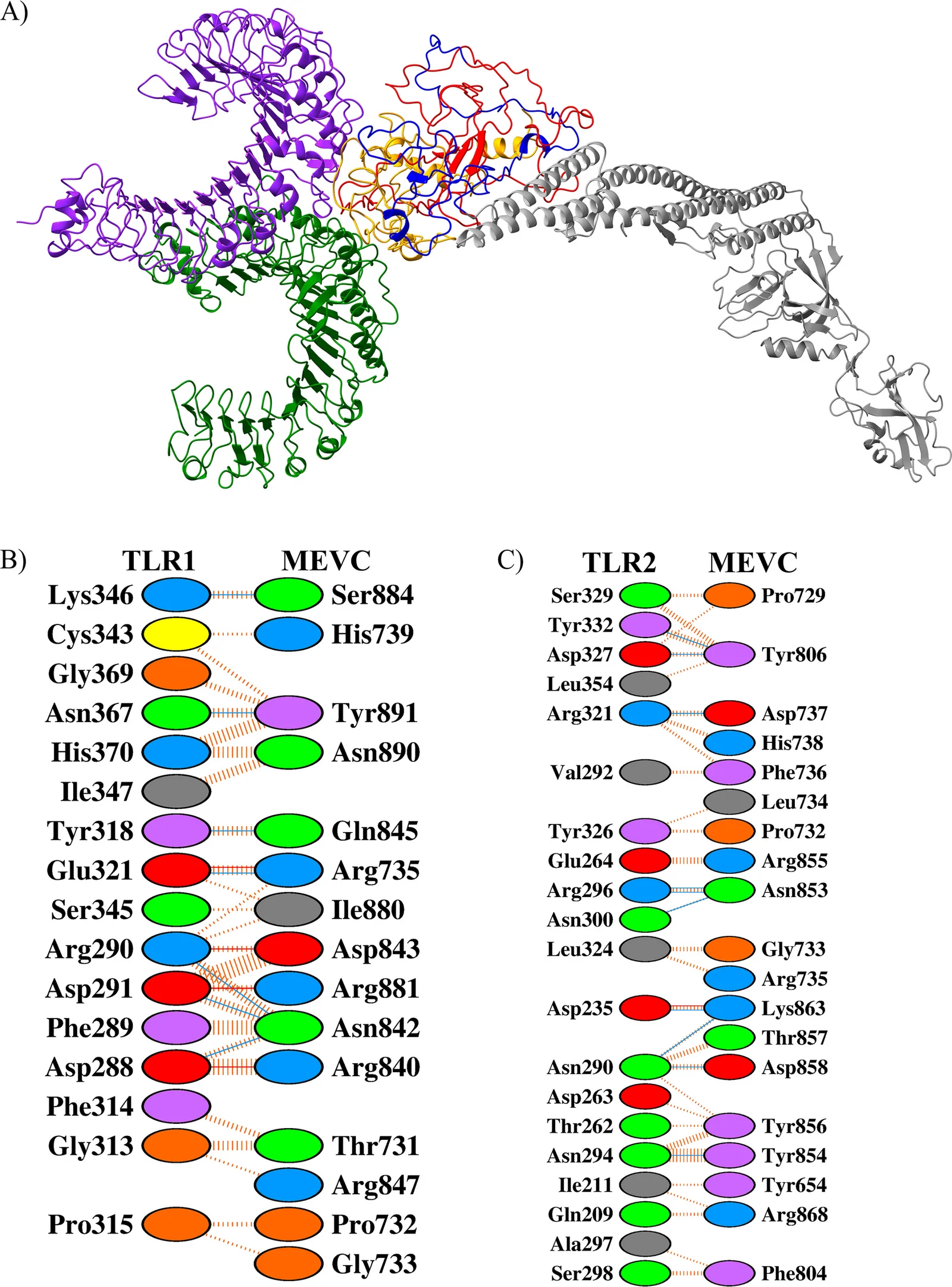
Molecular docking of multiepitope vaccine construct (MEVC) to toll-like receptor (TLR) 1/2 heterodimer. **A** ClusPro predicted 3D structures of MEVC docked to TLR1/2 heterodimer (Model 2) visualized by ChimeraX software with gray representing the adjuvant, blue the cytotoxic T cell epitope region, red the helper T cell epitope region, and orange the dual-purpose linear B cell epitope region. TLR1 is represented by green and TLR2 is indicated by purple Panels **B** and **C** are the interaction plots generated by PDBsum for MEVC (Chain C) to B) TLR1 (Chain B) and C) TLR2 (Chain A). In Panels **B** and **C**, solid red lines are salt bridges, solid yellow lines are disulfide bonds, solid blue lines are hydrogen bonds, and orange tick-marks are non-bonded contacts with longer tick marks indicating a greater number of contacts. Colored ovals represent: positive aa (blue), negative aa (red), neutral aa (green), aliphatic aa (gray), aromatic aa (purple), proline or glycine (orange), and cystine (yellow)

TLRs are cell surface or endosomal transmembrane proteins that serve a vital role in the activation of the innate immune system by binding to pathogen-associated molecular patterns (PAMPs). TLR1/2 was evaluated for *in silico* molecular docking prediction with the MEVC as this TLR complex binds bacterial lipoproteins, which are a subset of epitopes in the MEVC [137]. ClusPro Model 2 was the best ranked docking model where the MEVC docked TLR1/2 at the same region the tri-acylated lipopeptide docked in the reference (2X7Z) (Fig. 4A-C; Supplementary Table 7 – Additional File 2). ClusPro Model 2 had 32 residues interacting between the MEVC and TLR1/2, a center weighted score of −890.9 kcal/mol, and a lowest energy weighted score of −982.9 kcal/mol.

The MEVC epitopes projected to be involved in docking with TLR1 (*n* = 15 MEVC positions) were AEF06514.1 (FepA) in the Th region and AEF06873.1 (OmpF), AEF09863.1 (unnamed), and AEF10030.1 (LamB) in the DP-LBL region. The MEVC positions (*n* = 19) predicted to be involved in docking with TLR2 corresponded to residues in AEF06514.1 (FepA) and AEF10041.1 (TraF) in the Th region, and AEF06556.1 (PagP), AEF07866.1 (OmpS1, OmpC), and AEF09863.1 in the DP-LBL region (Supplementary Table 8 – Additional File 2). TLR2 was involved with one linker residue from the Th region (GPGPG).

#### Molecular dynamics

Molecular dynamics simulation was conducted to determine the stability and physical movements of the TLR 1/2 heterodimer-MEVC docked complex under physiological conditions. Prior to performing the molecular dynamics simulation (i.e. production run), shorter pre-production evaluations of potential energy, NVT, and NPT for the TLR1/2-MEVC complex were simulated to confirm that free energy, temperature, and pressure stabilized quickly (Supplementary Figs. 2A-E – Additional File 4; Supplementary Table 9 – Additional File 2). This indicates that the simulation environment was under equilibrium and energy minimization and was conducive for the prolonged molecular dynamic simulations.

**Fig. 5:**
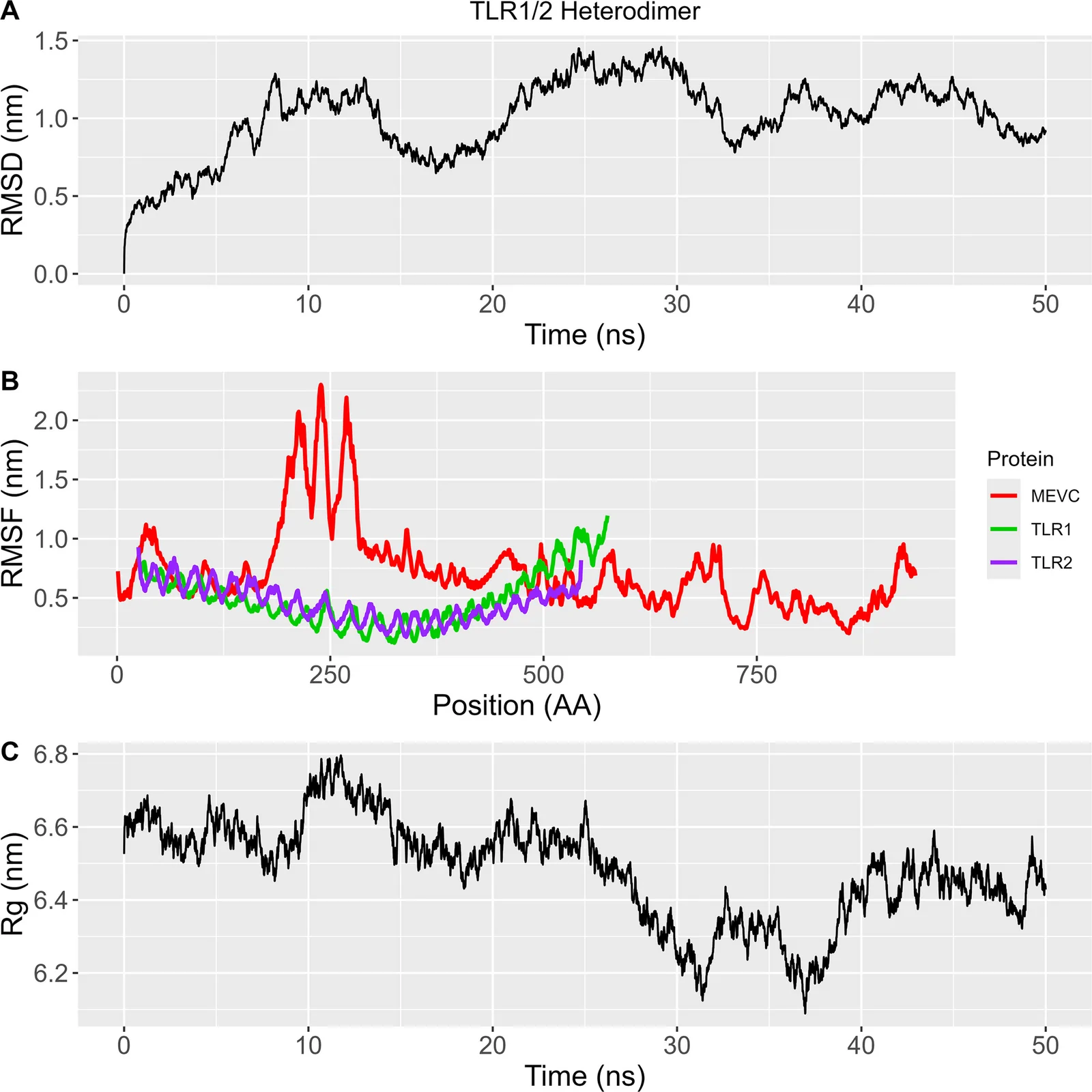
Molecular dynamics simulation of multiepitope vaccine construct (MEVC) docked to toll-like receptor 1/2 heterodimer. GROMACS 50 ns production run results are summarized in (**A**) RMSD (root mean square deviation) plot for the MEVC with TLR1/2 heterodimer, (**B**) RMSF (root mean square fluctuation) plot of MEVC with TLR1/2 heterodimer with color indicated for each chain (TLRs and MEVC), and (**C**) radius of gyration (Rg) plot of MEVC with TLR1/2 heterodimer

RMSD (an indication of structure stability) for the TLR1/2-MEVC complex revealed short periods of transient stabilization, but was still fluctuating by the end of the 50 ns simulation, suggesting the complex was flexible (Fig. 5A). TLR1 and TLR2 generally had smaller oscillations in RMSF across their residues (an indication of side-chain flexibility) compared to the MEVC (Fig. 5B). The range of RMSF values for the TLR1/2-MEVC complex was 0.201–2.30 nm for the MEVC, with the largest RMSF variations occurring in the MEVC from about positions 170 to 290 which correspond to the adjuvant residues farthest away from the MEVC binding site to the TLR1/2 heterodimer (Fig. 5B). The radius of gyration (a measure of structure compactness) for the TLR1/2-MEVC complex fluctuated but was stabilizing around 6.45 nm starting at 40 ns (Fig. 5C). The relative equilibration of the radius of gyration at approximately 40 ns indicates the complex was becoming compact and thus more stable. Overall, molecular dynamics simulations indicated that the MEVC is predicted to stably dock to the TLR1/2 heterodimer.

#### In silico immune simulations

**Fig. 6:**
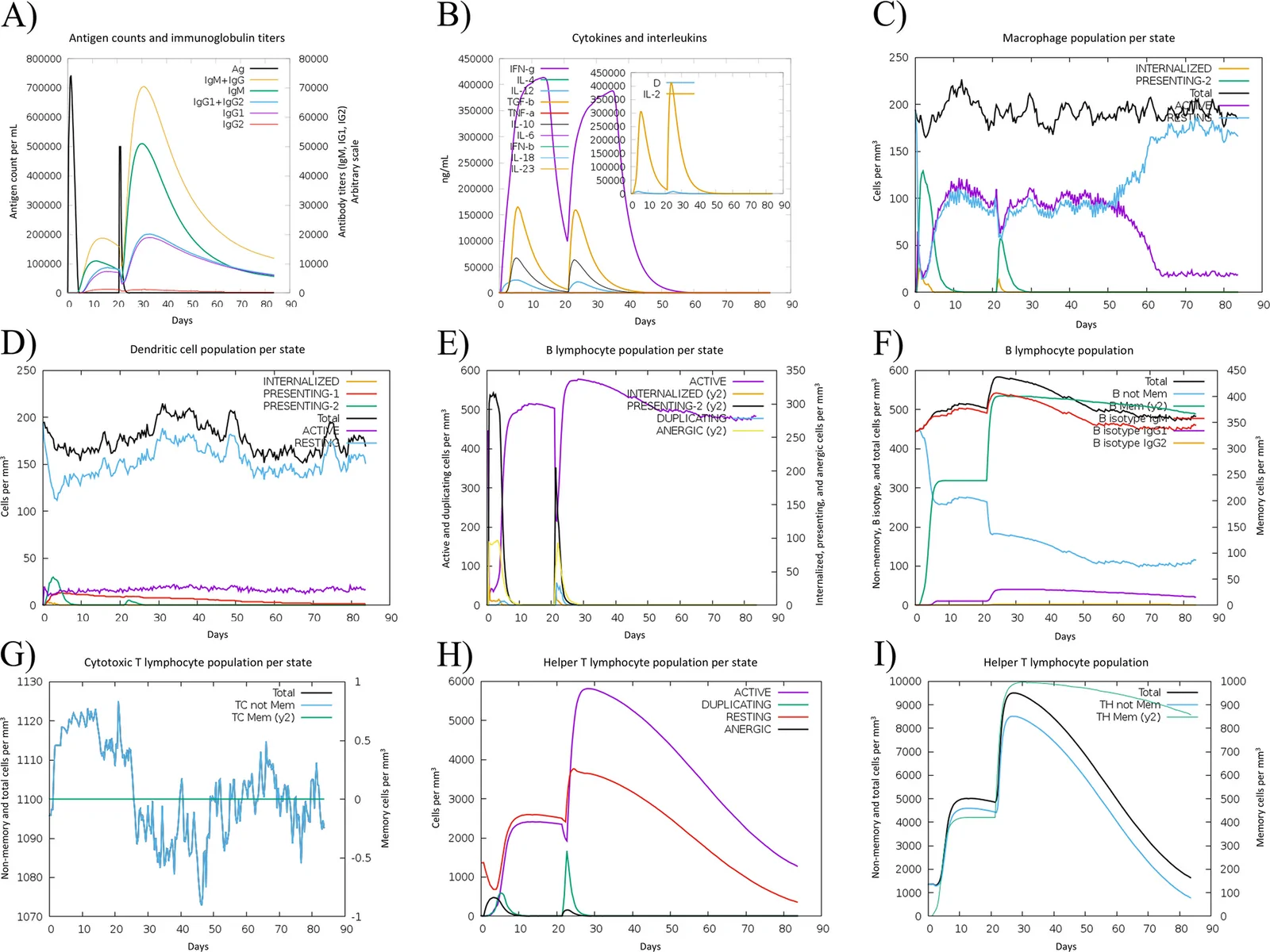
Immune simulation of MHCI HLA-B*41:04 and MHCII DRBI*14:45 alleles with the multiepitope vaccine construct (MEVC). C-ImmSim server simulation was run with injections of the MEVC occurring at Days 0 and 21. **A** MEVC antigen and immunoglobin levels, **B** production of cytokine and interleukins with Simpson index D, **C** macrophage population per state, **D** dendritic cell population per state, **E** B lymphocyte population per state, **F** B lymphocyte population, **G** cytotoxic T lymphocyte (TC) population, **H** helper T lymphocyte (TH) population per state, and **I** helper T lymphocyte population

*In silico* immune simulations were conducted to predict the potential for the MEVC to induce an immune response, although results from these human-centric simulations should be cautiously interpreted for chickens. C-ImmSim was initially performed with three pairs of alleles randomly selected from the chicken-like MHCI (*n* = 3) and MHCII alleles (*n* = 3) used to design the MEVC to determine if differences in predicted immune simulations occurred between the allele pairs. Immune simulation results from MHCI allele HLA-B*41:04 and MHCII allele HLA-DRBI*14:45 (Fig. 6) revealed similar trends as other random allele combinations (HLA-B*40:06 and HLA-DRBI*13:10 (Supplementary Fig. 3 – Additional File 4) as well as HLA-B*41:03 and HLA-DRBI*13:66 (Supplementary Fig. 4 – Additional File 4). Thus, additional combinations of the chicken-like alleles were not analyzed.

Simulated antibody responses (IgM > IgG1 + IgG2) were quicker and stronger to the second vaccine injection (booster at Day 21) than the first vaccine injection (Day 0), suggesting that under this *in silico* model, the MEVC was antigenic and successfully induced and boosted a humoral immune response (Fig. 6A, Supplementary Figs. 4A and 5A – Additional File 4). The stronger antibody response to the second injection also led to a narrower and diminished antigen peak, indicating the same dose of antigen was cleared more quickly upon secondary exposure. Although birds produce fewer immunoglobulin (antibody) isotypes than mammals, human predictions can still be translated as chicken IgM is homologous as the predominant isotype after initial exposure to a novel antigen, while chicken IgY is functionally equivalent to human IgG1 + IgG2 for the secondary immune response, as IgY is the ancestral isotype that evolved into IgG and IgE in mammals [138]. The most strongly activated cytokine was interferon gamma (IFNγ), which peaked shortly after each injection (Fig. 6B; Supplementary Figs. 4B and 5B – Additional File 4). Primarily secreted by activated Th cells, CTLs, and natural killer cells, IFNγ is pro-inflammatory and promotes cytotoxic activity and Th cell differentiation, which were also predicted to occur in the simulation [139].

Macrophage (Fig. 6C; Supplementary Figs. 4C and 5 C – Additional File 4), dendritic cell (Fig. 6D; Supplementary Figs. 4D and 5D – Additional File 4), and B cell (Fig. 6E; Supplementary Figs. 4E and 5E – Additional File 4) responses to the simulated injections suggested the MEVC would successfully initiate antigen presentation, with a rapid increase then decline of cells in an antigen presenting state after each exposure. Memory B cells increased after the first injection and rose again after the second exposure (Fig. 6F; Supplementary Figs. 4F and 5F – Additional File 4). The corresponding decrease in non-memory B cells indicates the differentiation from a naive to memory B cell state [140]. Non-memory CTLs peaked following the first MEVC injection but did not respond to a second vaccine injection (Fig. 6G; Supplementary Figs. 4G and 5G – Additional File 4). Th cells in the active, duplicating, and resting cell states as well as memory and non-memory Th populations were simulated to increase after each injection, with a higher peak after the booster (Fig. 6H and I; Supplementary Figs. 4H, 4I, 5H, 5I – Additional File 4). While non-memory Th cells decreased throughout the rest of the simulation to return to starting levels, most memory Th cells persisted to the end of the simulation. Taken together, the immune simulation results suggest the MEVC would be immunogenic due to the predicted establishment of both humoral and cellular immune responses, production of memory Th cells and B cells, and a stronger Th and B cell response to the second injection (booster vaccination) that resulted in faster clearance of the antigen compared to the initial vaccination.

### Phase V – Conservation of construct epitopes

To predict *in silico* if an immune response induced by the epitopes in the MEVC would protect against *Salmonella* isolates associated with human foodborne outbreaks, sequence homology of the MEVC epitopes was compared to 33,672 PulseNet isolates (representing 1,296 outbreak identifiers;135 named serovars; 167 unclassified isolates). PulseNet assigns a CDC outbreak identifier when a cluster of genetically related isolates is detected; for *Salmonella*, this requires at least 7–10 isolates within 0–10 cgMLST alleles of each other collected within a 60-day window [141]. The six human-relevant and poultry-associated serovars selected to design the MEVC represented 41% (13,891 isolates) of the PulseNet dataset: *S*. Enteritidis had the most isolates in the dataset (*n* = 6,239, 1 st), followed by *S.* Typhimurium (*n* = 3,438, 3rd), *S.* Hadar (*n* = 1,990, 4th), *S.* Infantis (*n* = 1,956, 5th), *S.* Uganda (*n* = 258, 20th), and *S.* Kentucky (*n* = 10, 94th) (Supplementary Table 10 – Additional File 2).

The number of *Salmonella* isolates that overlapped between the NCBI dataset downloaded for conserved homology to relevant serovars in Phase I (*n* = 90,800) and the PulseNet dataset (*n* = 33,672) was 1,850 isolates, representing 2% of the NCBI dataset and 5.5% of the PulseNet dataset; thus, influence of the PulseNet dataset in the initial selection of epitopes would have been minimal.

**Fig. 7:**
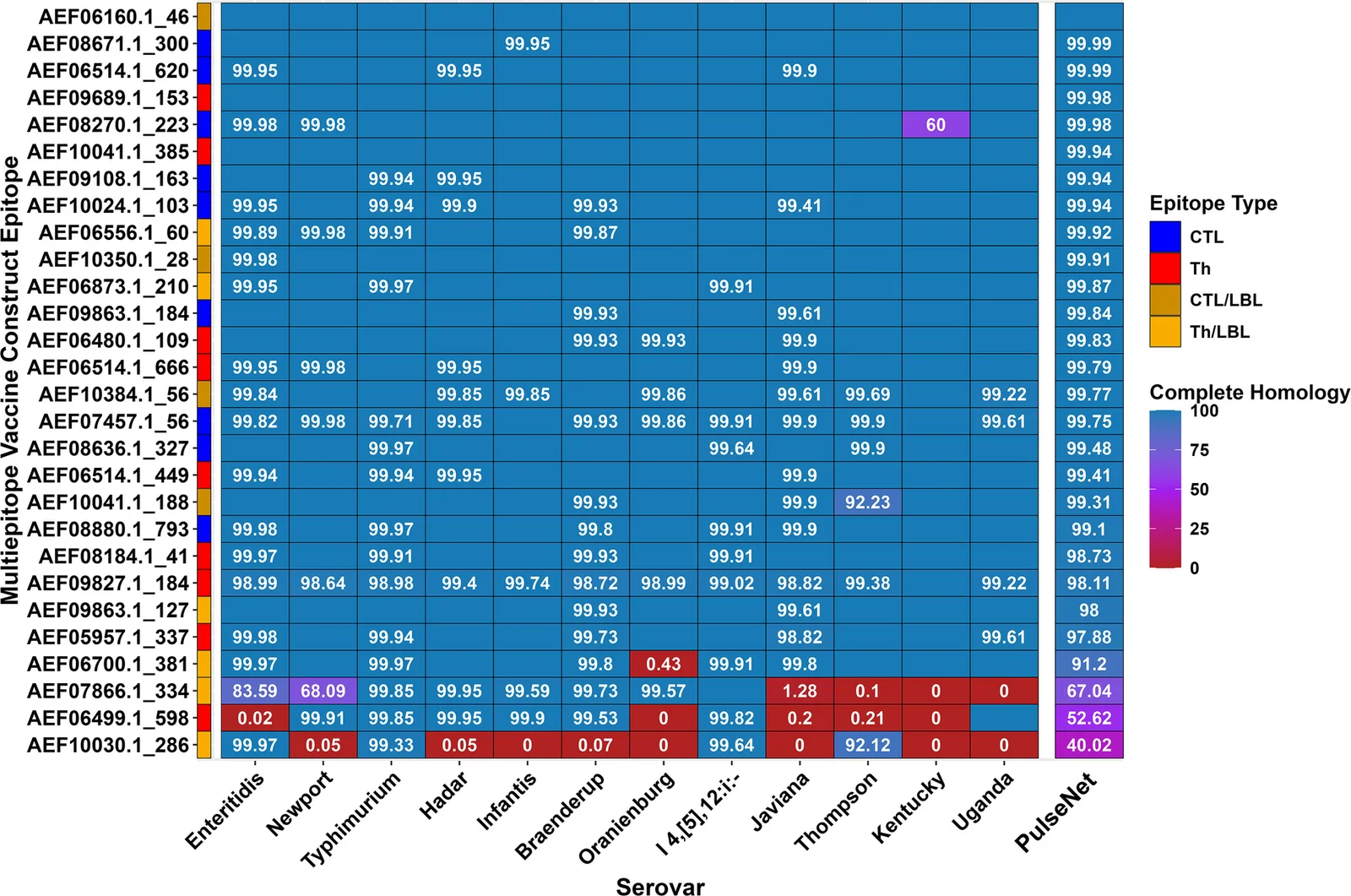
Alignment of multiepitope vaccine construct (MEVC) epitopes to PulseNet outbreak-associated proteomes from twelve *Salmonella* serovars. The color gradient (100% = dark blue, 50% = purple, 0% = dark red) in the heatmap illustrates the percentage of PulseNet outbreak-associated proteomes with complete homology (100% sequence identity and coverage) to each MEVC epitope (*n* = 28). Percentages are provided per select *Salmonella enterica* subspecies *enterica* serovar as well as for the overall PulseNet dataset (*n* = 33,672 isolates). Epitopes with complete homology to 100% of the proteomes do not have values within their cell. The selected serovars (*n* = 12) included the serovars used to design the MEVC (*S.* Enteritidis (*n* = 6,239), *S.* Typhimurium (*n* = 3,438), *S.* Hadar (*n* = 1,990), *S.* Infantis (*n* = 1,956), *S.* Kentucky (*n* = 10), *S.* Uganda (*n* = 258)) and the top ten serovars in terms of number of PulseNet isolates (*S.* Enteritidis, *S.* Newport (*n* = 4,328), *S.* Typhimurium, *S.* Hadar, *S.* Infantis, *S.* Braenderup (*n* = 1,490), *S.* Oranienburg (*n* = 1,380), *S.* I 4, [5], 12:i- (*n* = 1,118), *S.* Javiana (*n* = 1,014), *S.* Thompson (*n* = 956)). Colors on the left-hand side of the heatmap indicate the epitope type

PulseNet proteomes were aligned against each MEVC epitope to determine the percentage of proteomes with complete homology alignments with each MEVC epitope. In this study, complete homology is defined as 100% for both sequence identity (amino acids in the alignment that are the same between the PulseNet proteome and the epitope) and epitope coverage (percentage of epitope amino acids aligned to the proteome). To illustrate the trends in outbreak-relevant and MEVC-relevant serovars (*n* = 12), a subset of these results on a per-serovar basis is shown in Fig. 7.

Of the 135 serovars in the PulseNet dataset, three serovars had isolates with complete (100%) homology to the 28 MEVC epitopes including *S.* Baildon (*n* = 17 PulseNet isolates), *S.* Hvittingfoss (*n* = 7), and *S.* I -:i:1,2 (*n* = 4) (Supplementary Table 10 – Additional File 2). Three serovars including *S.* Typhimurium, *S.* I 4, [5],12:i:-, and Berta (*n* = 254; 21 st) had complete homology in 99% or more of the possible alignments (number of serovar proteomes in PulseNet database × 28 epitopes). An additional 54 serovars had complete homology in 90–99% of the possible alignments; these notably included *S.* Infantis, *S.* Hadar, *S.* Enteritidis, *S.* Uganda, *S.* Newport, *S.* Braenderup, and *S.* Thompson. Serovars of note with alignment percentages below 90% were *S.* Kentucky (87.86%), *S.* Oranienburg (89.24%), and *S*. Javiana (89.16%).

Sixteen MEVC epitopes had complete homology to all of the isolates within a serovar for ≥ 90% of the PulseNet serovars (Supplementary Table 10 – Additional File 2). Six MEVC epitopes had at least one alignment to every PulseNet serovar, while an additional eighteen epitopes had at least one alignment to 90% or more of the PulseNet serovars (Fig. 7; Supplementary Table 10 – Additional File 2). Overall, the sequence comparisons of the MEVC epitopes to serovars of the PulseNet dataset illustrate a high degree of epitope homology to not only the serovars used to design the MEVC, but also serovars associated with human foodborne illnesses and outbreaks.

Overall, 20 of the 28 MEVC epitopes had alignments with complete homology to ≥ 99% of the PulseNet proteomes, while 25 of the 28 MEVC epitopes had complete homology to ≥ 90% of the PulseNet isolates (Fig. 7; Supplementary Table 10 – Additional File 2). The three epitopes that did not pass the ≥ 90% threshold included: an Th/LBL epitope from LamB (AEF10030.1), an outer membrane maltoporin (39.93%); an Th epitope from an unnamed protein (AEF06499.1) annotated as an outer membrane esterase (52.49%); and an Th/LBL epitope from OmpS1 (AEF07866.1), an outer membrane porin (66.88%). Epitopes with complete homology alignments to the highest percentage of PulseNet isolates were: an DP-LBL epitope (CTL) from Skp (AEF06160.1), a periplasmic chaperone (99.76%); an CTL epitope from ProX (AEF08671.1), a glycine betaine transporter periplasmic subunit (99.75%); an CTL epitope from FepA (AEF06514.1), an outer membrane receptor (99.74%); and an Th epitope from PstS (AEF09689.1), a phosphate ABC transporter periplasmic substrate-binding protein (99.74%). Altogether, 89% of the MEVC epitopes, which were designed against six *Salmonella* serovars, exhibited complete homology (100% sequence identity across all amino acids within the epitope) to ≥ 90% of the outbreak-associated *Salmonella* isolates across 135 serovars in the PulseNet dataset, thereby suggesting the epitopes in the MEVC could potentially confer broad protection against multiple serovars in the various serogroups associated with human foodborne disease.

**Fig. 8:**
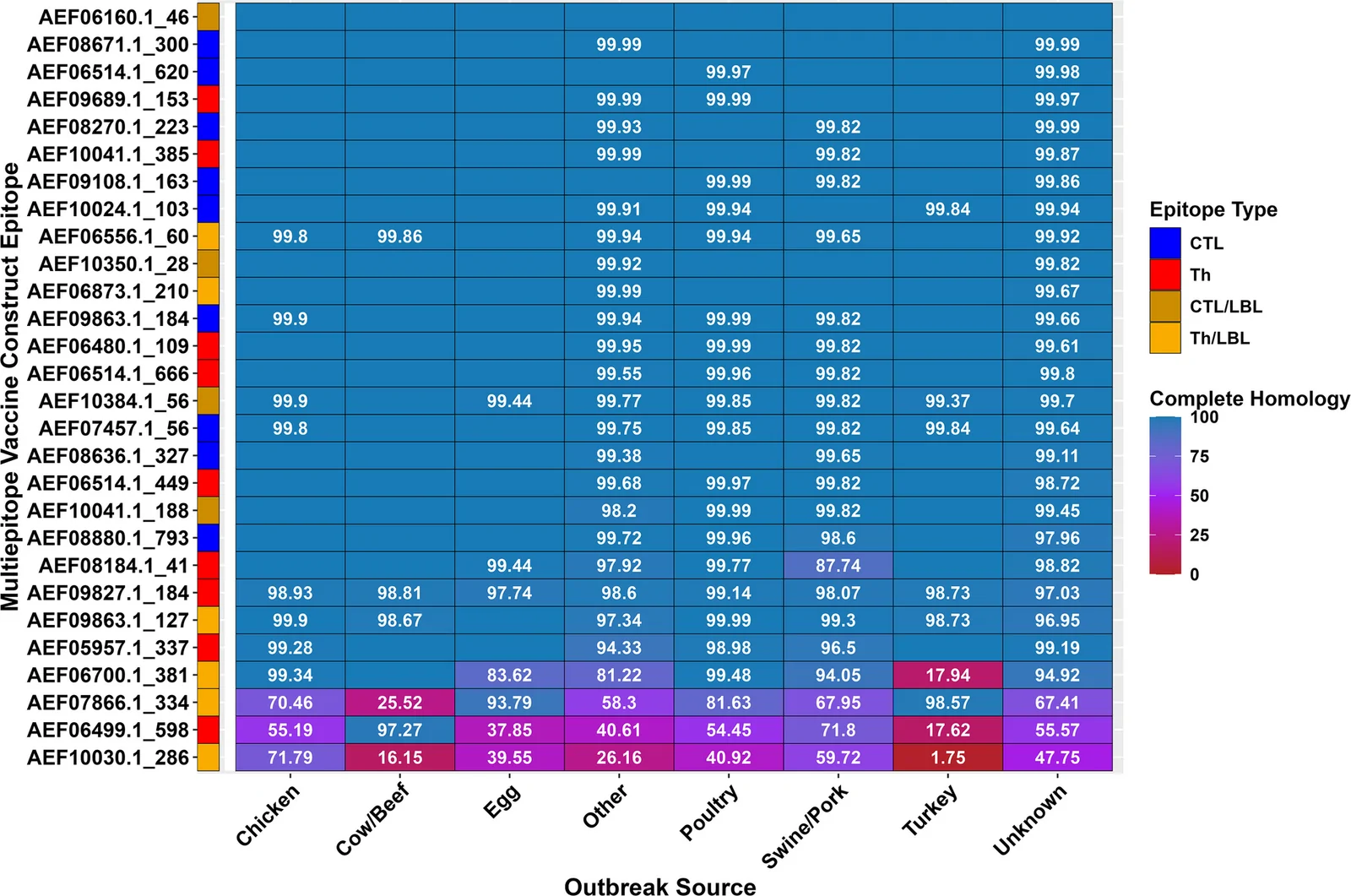
Alignment of multiepitope vaccine construct (MEVC) epitopes to PulseNet outbreak-associated proteomes by outbreak source. The color gradient (100% = dark blue, 50% = purple, 0% = dark red) in the heatmap illustrates the percentage of PulseNet outbreak-associated proteomes with complete homology (100% sequence identity and coverage) to each MEVC epitope (*n* = 28). Percentages are provided per specified outbreak source (*n* = 6) as well as an “Other” category that includes outbreak sources that were not the focus of this study (*n* = 8,865 isolates) and “Unknown” category that includes outbreak sources that were not identified (*n* = 13,162). The specified isolation sources included: poultry- (*n* = 6,880), chicken- (*n* = 1,957), turkey- (*n* = 630), cattle/beef- (*n* = 1,430), swine/pork- (*n* = 571), and egg-associated (*n* = 177) sources. Colors on the left-hand side of the heatmap indicate the epitope type

Homology between the 28 MEVC epitopes and the PulseNet dataset was also evaluated by isolation and outbreak sources. Isolation source details the sampling source from which the isolate was collected, whereas outbreak source details the known or attributed source for the outbreak, if it was identified. The most common isolation source from the PulseNet dataset was humans (*n* = 31,012) (Supplementary Table 11 – Additional File 2). Other isolation sources of interest included chicken (*n* = 726), turkey (*n* = 377), cattle/beef (*n* = 270), swine/pork (*n* = 260), and eggs (*n* = 6). All six isolation sources of interest had complete homology to ≥ 90% of all possible alignments (i.e. number of serovar proteomes in PulseNet database × 28 epitopes), ranging from 95.51% of chicken-associated to 91.95% of turkey-associated isolates (Supplementary Table 11 – Additional File 2). Complete homology to 99% or more of all chicken-, turkey-, and egg-associated isolate sources was observed for 24 of the 28 MEVC epitopes (Supplementary Fig. 5 – Additional File 4), followed by cattle/beef- (23 of 28), human- (20 of 28), and swine/pork-associated (20 of 28) isolate sources. The most common outbreak source of interest was poultry (*n* = 6,880), with the addition of further samples designated as chicken (*n* = 1,957), turkey (*n* = 630), or egg (*n* = 177) outbreak sources (Supplementary Table 12 – Additional File 2). The non-poultry-related outbreak source of interest with the most isolates was cattle/beef (*n* = 1,430) followed by swine/pork (*n* = 571). Twenty-four out of 28 MEVC epitopes had complete homology to 99% or more of all isolates from chicken- and poultry-associated outbreaks followed by egg- (23 of 28), cattle/beef- (23 of 28), turkey- (22 of 28), and swine/pork-associated outbreaks (20 of 28) (Fig. 8). Complete homology to 99% or more of isolates from outbreaks not associated with sources of interest (*n* = 13,132) or from outbreaks with undefined sources (*n* = 8,865) was noted for 19 of the 28 MEVC epitopes (Fig. 8; Supplementary Table 12 – Additional File 2). Isolates from chicken-associated outbreaks had the most complete homology alignments to all possible alignments (96.22%), while turkey-associated outbreak isolates had the lowest (90.44%). Unlike the per serovar analysis, every MEVC epitope had at least one complete homology alignment to at least one isolate from each isolation and outbreak source of interest. Thus, while the chicken-targeted MEVC was designed based on human-relevant and poultry-associated *Salmonella* serovars using chicken-like MHC alleles, high alignment of the MEVC to PulseNet outbreak-associated isolates from other sources of interest suggests the potential use of the MEVC to reduce *Salmonella* in other food-producing animals (turkey, swine, or cattle) or food-related outbreaks in humans.

## Discussion

Reverse vaccinology (RV) was implemented as an *in silico* approach to address a major hurdle and limitation of current *Salmonella* vaccines: effective cross-protection against the > 2600 serovars of *Salmonella*. Our RV pipeline avoided the immunovariable and immunodominant LPS molecules and flagellar proteins, and instead focused on conserved outer membrane, periplasmic and extracellular proteins of human-relevant and poultry-associated *Salmonella* serovars from serogroups B-E.

Numerous tools exist to inform and predict each stage of the RV pipeline [55] and have been employed to investigate a range of bacteria and viruses, including *Salmonella* [13, 14, 33, 41, 45, 46, 54, 56, 84, 85, 117–119, 142–150]. Tool choices of our RV pipeline were based on selective curation of tools and filters from the RV literature, the relative usage of tools at each step, and availability of non-server-based tools that allow for customization [13, 14, 45, 56, 65, 119]. A major limitation with current RV tools is that most tools are designed for use in a human host. Our workflow was modified in an effort to address this issue, but careful interpretation for poultry should be taken with some of the predictive results, in addition to the inherent limit of *in silico* simulations optimally translating to *in vivo* outcomes.

Some RV approaches use *a priori* selection of specific and logically chosen antigenic or virulent proteins such as OmpA, OmpC, OmpF, OmpS, SopB, SopF, SseB, SthA, FilC, FlgK, or DnaK [84, 142, 144, 145, 151]. To avoid limiting the discovery of new or potentially cross-protective targets, the complete chromosomic proteome of *S.* Typhimurium strain UK-1 was used in Phase I (Subtractive Proteomics) to screen proteins for desirable traits, resulting in 101 proteins. While some previous studies limited their analysis to outer membrane proteins due to their immunological importance, this study expanded protein interrogation to include periplasmic and extracellular proteins that may also be relevant to pathogen recognition by the immune system [35, 41, 152].

An ideal vaccine should elicit protective immunological memory through both cell-mediated and humoral immune response mechanisms; thus, Phase II predicted CTL, Th, and LBL epitopes from the Phase I identified proteins. While most of the RV steps used in Phase II (Epitope Identification and Filtering) were similar to other RV studies, the pipeline was optimized with the following modifications. First, chicken-like human MHC alleles were selected based on Valdivia-Olarte et al. (2015) and other poultry-focused RV studies for CTL (MHCI) and Th (MHCII) epitope predictions [47, 56, 147]. Humans rely on the combined peptide repertoires of multiple MHCI and MHCII proteins, while chickens only possess two classical MHCI (BF) and MHCII (BLB) genes, of which only a single loci is dominantly expressed in most tissues [153]. Although the number of MHC alleles is low in commercial chickens, those alleles are typically able to bind a wide variety of peptides [153], providing the capacity for a broader peptide repertoire from a single chicken MHC gene than observed in humans as well as immune recognition of most common disease antigens using that single loci [154, 155]. Although the peptide repertoires of the chicken-like human MHCI (*n* = 3) and MHCII (*n* = 4) alleles likely do not encompass the same range of epitopes recognized by the commercial chicken line used in the analysis by Valdivia-Olarte et al., these chicken-like human loci are more likely to have shared peptide affinities and allow selection of epitopes that translate to chickens.

The second modification to Phase II attempted to more robustly address broad protection by employing a sequence conservation requirement. Other studies identified highly conserved proteins through various methods [54] including: selection for proteins with 90% similarity among 16 *Clostridium perfringens* isolates [41] or 98% conservation among 182 *Salmonella* Gallinarum Pullorum clinical isolates [146]; identification of core genes using pangenomic analysis of 119 *S.* Typhi reference genomes [156] or 173 *Campylobacter jejuni* isolates [157]; and comparison of the core genomes from 30 *Salmonella* strains representing *Salmonella bongori*, *Salmonella enterica* subspecies *arizonae*, and 15 serovars of *Salmonella enterica* subspecies *enterica* [46]. Another study evaluated cross-homology by assessing their selected epitopes for homology against the serovars that the MEVC was designed against with BLAST analysis: *S.* Typhi, *S*. Paratyphi A, *S*. Typhimurium, and *S.* Enteritidis [117]. The present study expanded upon this concept by aligning Phase II epitopes against all NCBI assemblies of the human-relevant and poultry-associated serovars *S*. Typhimurium, *S*. Enteritidis, *S*. Infantis, *S*. Kentucky, *S*. Hadar, and *S*. Uganda (*n* = 90,800). Testing for cross-homology at the epitope level more efficiently utilizes computational time and power by allowing for the analysis of a large number of proteomes, compared to testing for cross-homology at the protein level via pangenome analysis as is used in previous studies.

The third modification in Phase II employed dual-purpose LBL epitopes, i.e. Th and CTL epitopes that also represented LBL epitopes, to efficiently use construct space. In humans, LBL epitopes have a range of 8 to 25 residues, thus the included LBL epitopes were biased towards the shorter end of the natural length spectrum (9- or 15-mers for CTL or Th epitopes, respectively). MEVC inclusion of epitopes for B lymphocytes can be challenging because 90% are conformational not linear, and while both humans and chickens use V(D)J gene arrangement and somatic hypermutation, chickens have far less germline encoded variation in their immunoglobulin heavy and light chains than humans and instead create immunoglobulin diversity by incorporating sequences from pseudogenes through a process called somatic gene conversion [158]. The literature is lacking on how to address the differences between human and chicken immunoglobulin generation to appropriately perform an LBL epitope prediction. Thus, dual-purpose LBL epitopes were identified and incorporated into the MEVC to ensure value with the inclusion of overlapping CTL or Th epitopes.

In Phase III, Construct Design, the proposed first draft of MEVC sequence incorporated 28 epitopes and epitope-type-associated linkers into the epitope region (441 aa). While the size of this region is in line with previous literature (171–678 aa), and various *in silico* tests suggest the MEVC as a whole is stable, additional modifications such as logically shuffling epitopes or reducing the size of the epitope region may be required to ensure better stability once actually synthesized [13, 46]. Sixteen of twenty-four proteins represented by the MEVC epitopes were identified as having roles in host colonization or as vaccine candidates in the literature which suggests that these proteins are expressed *in vivo* and led to stimulation of the immune response. For example, deletion of genes encoding certain *Salmonella* outer membrane porins (OmpC and OmpF), which play a major role as passive diffusion channels for small molecules, nutrients, antibiotics, and toxic salts, have been shown to be attenuated for virulence in mice [136]. Studies exploring porins as vaccine targets have shown that these highly immunogenic proteins activate the innate and adaptive immune responses [121, 127, 159–161]. Recombinant OmpF (AEF06873.1) was immunoprotective against *S.* Enteritidis challenge in chickens [121], and combined immunization with OmpA, OmpC (AEF08184.1; ~ AEF07866.1 aka OmpS1), and OmpF was immunoprotective against *S. enterica* subsp. Typhi (PTCC 1609) in mice [127]. OmpC, OmpD, OmpF, and OmpL (AEF09827.1 aka YshA) were shown via RV to contain both CTL and Th epitopes [13, 117, 118, 162]. A recombinant form of the oligogalacturonate-specific porin OmpL was immunoprotective in murine models following *S.* Typhimurium infection, resulting in reduced pathogen load, improved host survival, and induction of anti-OmpL serum with bactericidal activity against *S.* Typhi, *S.* Paratyphi, *S.* Paratyphi B, and *S.* Paratyphi C [126].

Another transport protein incorporated in the MEVC and identified in the literature as an immunoprotective protein was PstS (AEF09689.1), a periplasmic substrate-binding protein responsible for transferring phosphate to the bacterial cytoplasm through the phosphate ABC transporter [163]. PstS was identified as a vaccine candidate in a recent RV study against *Salmonella* Gallinarum Pullorum R51; vaccination increased chick survival from 31.25% to 75% following R51 challenge [41]. LamB (AEF10030.1), a maltose and maltodextrin transporter, and surface receptor for bacteriophages, was immunogenic and immunoprotective against *Vibrio* spp. and *Salmonella* Paratyphi A CMCC 50973 in recombinant protein studies in zebrafish and mice, respectively [124, 125, 128]. FepA (AEF06514 and AEF08636.1) is a TonB-dependent transporter (TBDT) for siderophores enterobactin and 2,3-dihydroxybenzoylserine. FepA and other *Salmonella* TBDTs CirA and IroN have been explored as vaccine candidates due to their conserved surface exposure and role in virulence [164]. In another RV study, FepA was immunogenic in ELISA using antiserum from mice immunized with purified FepA [116]. FepA has high sequence similarity to IroN whose epitopes in a multiepitope vaccine led to strong humoral, cellular, and mucosal immune responses after nasal administration in a mouse model of extraintestinal pathogenic *E. coli* peritonitis [165, 166]. Siderophore receptor and porin proteins have been incorporated into commercially available vaccines including the Vaxxon™ SRP® *Salmonella* (formerly *Salmonella* Newport Bacterial Extract) (Vaxxinova, Niijmegen, Netherlands) developed for sheep and cattle [167, 168].

Several epitopes in the MEVC were associated with fimbriae-related proteins, including BcfC (AEF05957.1; FimD), a fimbrial usher protein involved in the export of the Bcf fimbria subunits through the usher channel facilitating bacterial adhesion to abiotic surfaces [169, 170]. The *bcfC* gene is a common PCR target for *Salmonella* virulence studies and its conserved PapC N-terminal domain has been suggested as a vaccine target [170, 171]. Immunization of mice with a cocktail of multiple fimbria subunits, including BcfA and SthA (see below), individually fused to glutathione S-transferase (GST) reduced *S.* Typhimurium fecal shedding following challenge in comparison to the GST control mice [123]. These data in combination with increased murine serum IgG and fecal IgA levels suggest these proteins are immunogenic and immunoprotective [123]. SthA (AEF10384.1; major fimbrial subunit protein), FimD, and StdB (AEF08880.1; fimbrial usher protein) were identified via RV to have highly antigenic epitopes in *S*. Typhi (FimD, SthA, StdB), *Stenotrophomonas maltophilia* (FimD), and *Enterobacter cloacae* (FimD) [13, 84, 114, 115, 119, 120].

Besides OmpF and OmpC discussed above, five additional MEVC proteins were described as potential vaccine targets in the literature due to virulence attenuation after genetic disruption of these genes in various bacterial species. A gene knockout mutation in the periplasmic molecular chaperone Skp (AEF06160.1; OmpH) results in virulence attenuation of *S*. Typhimurium in a murine model [129], while a cyclic synthetic peptide of the OmpH loop 2 confirmation is immunoprotective and immunogenic in a chicken challenge study with *Pasteurella multocida* strain X-73 [122]. PagP (AEF06556.1) through palmitoylation of the lipid A component of LPS decreases pathogen susceptibility to cationic antimicrobial peptides (CAMP) including α-helical peptides produced by the host, and helps *Salmonella* avoid the host immune system by reducing the ability to activate the host TLR4 pathway [172, 173]; PagP has been a knockout target in multiple attenuated vaccine studies with varying degrees of success [130, 131]. YncD (AEF07457.1; PqqU) is a TBDT for pyrroloquinoline quinone (PQQ), a quinone redox cofactor for oxidative enzymes [174]. Deletion of *yncD* results in attenuated *Salmonella* strains that are immunoprotective against wild-type challenge of *S.* Typhi and *S.* Paratyphi A in mice models [133, 135]. Loss of the histidine transport protein HisJ (AEF08270.1) results in deleterious effects on virulence traits such as biofilm formation, adhesion, invasion, and flagellar shedding, resulting in virulence attenuation of *Aeromonas veronii* that has potential for live vaccine development in aquaculture [132]. Deletion of YraP (AEF09108.1), a divisome-associated lipoprotein, leads to loss of cell envelope integrity and virulence attenuation of *S.* Typhimurium in a murine model [134].

Eight proteins that provided epitopes for the MEVC in the present RV pipeline have not been previously described or evaluated as vaccine targets. TraF (AEF10041.1) is a protease required for mobilization of conjunctive plasmids and donor-specific bacteriophages [175]. AEF10350.1 had high sequence identity to *Salmonella* proteins annotated as OsmY, an osmotic stress response protein. ProX (AEF08671.1) is a glycine betaine/L-proline ABC transporter substrate-binding protein. AEF06480.1 (fimbriae assembly protein), AEF06499.1 (autotransporter domain-containing esterase), AEF06700.1 (acyl-CoA thioester hydrolase), AEF10024.1 (YjbH domain-containing protein), and AEF09863.1 (oligogalacturonate-specific porin KdgM family protein) were functionally hypothesized but not annotated as named proteins in UK-1 nor did they share high identity (≥ 98%) to other *Salmonella* proteins. The identification of ten epitopes from unnamed proteins (*n* = 5) or proteins not previously explored in vaccine development (*n* = 3) corroborates the use of the whole proteome for identification of potentially novel vaccine targets.

In Phase IV, Assessment of Construct Properties, the MEVC was examined for appropriate physiochemical properties, tertiary structure was predicted, and a reproducible method was developed to select a refined tertiary model for molecular docking and dynamics simulations; the method employed ranking the scores of the three main GalaxyRefine metrics to select a refined tertiary structure model that balanced similarity to the original model (RMSD and GHT-HS) while also improving upon the similarity to known protein crystal structures and minimizing spatial clashing between residues (MolProbity).

*In silico* molecular docking of the MEVC was performed with the TLR1/2 heterodimer due to its relevance in the chicken immune response to bacterial pathogens and to certain epitopes in the MEVC. Although ClusPro suggests choosing the model representative of the cluster with the most members, this pipeline first prioritized docking models that predicted the MEVC would bind TLR1/2 in the same region that the reference ligand also binds to TLR1/2 in the reference crystal structure. This comparison adds an additional physiologically motivated step that helps ensure that the selected model is consistent with the known biological interactions of the specific ligand and receptors being tested. A biologically relevant TLR1/2 heterodimer docking model was selected based upon the modified pipeline and further evaluated for molecular dynamics. A potentially negative consequence of this method is that the most stable structures (i.e. those with the most cluster members or lowest energy) were not chosen because the MEVC did not dock to the expected ligand-binding region. This may have affected the molecular dynamics simulations by providing a less stable receptor-ligand complex model than was possible. Molecular dynamics was conducted as close to physiological conditions for chickens as could be found in the literature to continue to incorporate host relevance into the pipeline. The predicted nonequilibrium state of the TLR1/2-MEVC complex may potentially cause instability *in vivo*, furthermore, the MEVC may bind other TLRs or other immune receptor classes not tested.

Immune simulation with the chicken-like human MHC alleles used in epitope prediction yielded the expected cellular and humoral immune responses to the MEVC, with stimulation of antigen presenting cells, and memory B and Th cells in response to both injections, as well as prediction of boosted immune responses (smaller antigen peak, greater production of immunoglobulins, larger Th response) after the second injection [13]. Future *in vivo* or *in vitro* studies to evaluate the MEVC in chickens will incorporate transcriptomic and immunological techniques to measure alterations in the host transcriptional and immune responses to confirm this predicted immunogenicity.

The addition of Phase V incorporated a novel testing process whereby sequence homology of the MEVC epitopes was compared against PulseNet outbreak-associated assemblies, a dataset representing 135 *Salmonella* serovars, to test how well a MEVC designed from a limited number (*n* = 6) of human-relevant, poultry-associated *Salmonella* serovars extends to other serovars of clinical importance. Twenty-five of the 28 epitopes exhibited complete homology to 90% or more of the outbreak-associated *Salmonella* isolates. Considering that *Salmonella* colonization of poultry tends to be dominated by 15 or fewer serovars each year, the sequence conservation with the 135 PulseNet outbreak-associated serovars corroborates the initial selection pipeline for the MEVC epitopes. While the RV pipeline was designed to generate a MEVC targeted at chickens, delineating the PulseNet dataset by isolation and outbreak sources also demonstrated the MEVC had high homology against other food animals of interest (cattle, turkey, and swine) as well as isolates that caused outbreaks in humans with food animal-related sources. Combined with the published evidence that 16 out of 24 MEVC proteins are immunogenic, immunoprotective, and/or suggested targets for vaccine development, the PulseNet testing step provides confidence that the MEVC has a high degree of cross-homology against various *Salmonella* serovars/serogroups with promise for cross-reactivity, immunoprotection, and applications beyond poultry.

## Conclusion

This study used a modified RV pipeline to computationally screen for homologous epitopes from conserved *Salmonella* proteins to design a prospective MEVC with potential broad protection. The MEVC candidate incorporated 28 antigenic, non-toxic, hydrophilic epitopes from 24 proteins with 100% identity across six human-relevant and chicken-associated serovars representing *Salmonella* serogroups B-E. With the addition of a proprietary *Salmonella* flagellin as an adjuvant and linkers, the MEVC was predicted to have high antigenicity, non-allergenicity, non-toxicity, as well as appropriate stability, thermostability, and solubility. Using *in silico* tools, the proposed tertiary structure stably docked to a TLR1/2 heterodimer via molecular dynamics and the MEVC sequence was predicted to instigate a strong humoral and cellular immune response. The whole proteome approach was endorsed by not only identifying previously recognized proteins with immunogenic, immunoprotective and/or virulence attenuation properties (*n* = 16), but also proteins (*n* = 8) not previously identified as vaccine targets in the literature. Cross-homology of the MEVC was confirmed by demonstrating that 25 out of 28 MEVC epitopes had complete sequence identity to > 90% of human outbreak-associated isolates within a PulseNet dataset representing 135 outbreak-associated *Salmonella* serovars across five food-animal outbreak sources. Collectively, the RV approach provides a foundational *in silico* vaccine design pipeline that supports further creation and *in vivo* or *in vitro* evaluation of a potential MEVC for immune stimulation and cross-protection against the foodborne pathogen *Salmonella* in poultry.

## Supplementary Information

Additional file 1: Expanded technical notes describing theory and details associated with tools used in this study.

Additional file 2: Additional Tables relevant to manuscript.

Additional file 3: Additional Tables with accession numbers for proteins, assemblies, and reads used during analyses.

Additional file 4: Additional Figures relevant to manuscript.

## Data Availability

The protein sequences with alignments to query proteins during Phase I analysis steps for similarity to other *Salmonella* proteins (Accession Numbers Table 1 – Additional File 3), little to no homology to potential hosts (Accession Numbers Table 2 – Additional File 3), and sequence conservation to select *Salmonella* serovars (Accession Numbers Table 3 – Additional File 3) in the current study are available from the NCBI Protein Database (https://www.ncbi.nlm.nih.gov/protein/). The GenBank assemblies (Accession Numbers Table 4 – Additional File 3) tested for epitope sequence conservation during Phase II in the current study are available from the NCBI Assembly Database (https://www.ncbi.nlm.nih.gov/home/genomes/). The metadata that supports the epitope sequence conservation against outbreak-associated isolates analyses and findings of this study is available from United States Centers for Disease Control and Prevention, but restrictions apply to the availability of these data and is only available upon formal reasonable request from PulseNet. The reads (Accession Numbers Table 5 – Additional File 3) used to create the assemblies for this step are available from the NCBI Sequence Read Archive (https://www.ncbi.nlm.nih.gov/sra). The datasets and metadata generated during the current study are available from the corresponding authors on reasonable request or can be generated by using the scripts on GitHub on the above datasets (https://github.com/USDA-FSEPRU/rev_vacc_salm_mevc.git).

## Abbreviations

LPS: Lipopolysaccharide
NTS: Non-typhoidal *Salmonella*
CDC: Centers for Disease Control and Prevention
RV: Reverse vaccinology
*S*. Example: *Salmonella**enterica* Subspecies *enterica* serovar Example
ML: Machine learning
SSP: Similar *Salmonella* proteins
E-value: Expect value
CTL: Cytotoxic T lymphocyte
Th: Helper T lymphocyte
LBL: Linear B lymphocyte
MHC: Major histocompatibility complex
HLA: Human leukocyte antigen
IEDB: Immune Epitope Database
SVM: Support vector machines
GRAVY: Grand average of hydropathy
CBL: Confirmational B lymphocyte
DP-LBL: Dual-purpose linear B lymphocyte
MEVC: Multiepitope vaccine construct
TAP: Transporter-associated with antigen processing
TLR: Toll-like receptor
GDT-HA: Global Distance Test-High Accuracy
RMSD: Root mean square deviation
LRR: Leucine rich region
OPLS-AA/L: Optimized Potential for Liquid Simulation All Atom LMP2
SPC/E: Extended simple point charge model
NVT: Constant number of particles, volume, and temperature
NPT: Constant number of particles, pressure, and temperature
RMSF: Root mean square fluctuation
SRA: Sequence Read Archive
IG: Immunogenic
AT: Attenuation target
IP: Immunoprotective
PAMP: Pathogen-associated molecular pattern
IFNγ: Interferon gamma
TBDT: TonB-dependent siderophore transporter

## References

[1] Shi C, Singh P, Ranieri ML, Wiedmann M, Moreno Switt AI. Molecular methods for serovar determination of *Salmonella*. Crit Rev Microbiol. 2015;41(3):309–25. 24228625 10.3109/1040841X.2013.837862

[2] Brenner FW, Villar RG, Angulo FJ, Tauxe R, Swaminathan B. *Salmonella* nomenclature. J Clin Microbiol. 2000;38(7):2465–7. 10878026 10.1128/jcm.38.7.2465-2467.2000 PMC86943

[3] Plumb IF, Patricia; Bruce, Beau. CDC Yellow Book: Salmonellosis, Nontyphoidal: Centers for Disease Control and Prevention. 2024. Available from: https://wwwnc.cdc.gov/travel/yellowbook/2024/infections-diseases/salmonellosis-nontyphoidal.

[4] CDC. *Salmonella*. 2023. Available from: https://www.cdc.gov/salmonella/index.html. Accessed 21 Nov 2023.

[5] Hoffmann S, White AE, McQueen RB, Ahn JW, Gunn-Sandell LB, Scallan Walter EJ. Economic burden of foodborne illnesses acquired in the United States. Foodborne Pathog Dis. 2025;22(1):4–14. 39354849 10.1089/fpd.2023.0157

[6] Interagency Food Safety Analytics Collaboration. Foodborne illness source attribution estimates for *Salmonella*, *Escherichia coli* O157, and *Listeria monocytogenes* — United States, 2021. Centers for Disease Control and Prevention (CDC), the U.S. Food and Drug Administration (FDA), and the U.S. Department of Agriculture’s Food Safety and Inspection Service (USDA-FSIS). 2023. Available from: https://www.cdc.gov/ifsac/php/data-research/annual-reports/index.html.

[7] Mkangara M. Prevention and control of human *Salmonella enterica* infections: an implication in food safety. Int J Food Sci Nutr. 2023;2023(1):8899596. 10.1155/2023/8899596 PMC10506869 37727836

[8] Ruvalcaba-Gómez JM, Villagrán Z, Valdez-Alarcón JJ, Martínez-Núñez M, Gomez-Godínez LJ, Ruesga-Gutiérrez E, et al. Non-antibiotics strategies to control *Salmonella* infection in poultry. Animals. 2022;12(1):102. 35011208 10.3390/ani12010102 PMC8749512

[9] Gu G, Strawn LK, Zheng J, Reed EA, Rideout SL. Diversity and dynamics of *Salmonella enterica* in water sources, poultry litters, and field soils amended with poultry litter in a major agricultural area of Virginia. Front Microbiol. 2019;10:2868. 31956319 10.3389/fmicb.2019.02868 PMC6951424

[10] Bearson BL, Brunelle BW. Fluoroquinolone induction of phage-mediated gene transfer in multidrug-resistant *Salmonella*. Int J Antimicrob Agents. 2015;46(2):201–4. 26078016 10.1016/j.ijantimicag.2015.04.008

[11] Bearson BL, Allen HK, Brunelle BW, Lee IS, Casjens SR, Stanton TB. The agricultural antibiotic carbadox induces phage-mediated gene transfer in *Salmonella*. Front Microbiol. 2014;5:52. 24575089 10.3389/fmicb.2014.00052 PMC3920066

[12] Crouch CF, Nell T, Reijnders M, Donkers T, Pugh C, Patel A, et al. Safety and efficacy of a novel inactivated trivalent *Salmonella enterica* vaccine in chickens. Vaccine. 2020;38(43):6741–50. 32888739 10.1016/j.vaccine.2020.08.033

[13] Chand Y, Singh S. Prioritization of potential vaccine candidates and designing a multiepitope-based subunit vaccine against multidrug-resistant *Salmonella* Typhi str. CT18: a subtractive proteomics and immunoinformatics approach. Microb Pathog. 2021;159:105150. 34425197 10.1016/j.micpath.2021.105150

[14] Beikzadeh B. Immunoinformatics design of multi-epitope vaccine using OmpA, OmpD and enterotoxin against non-typhoidal salmonellosis. BMC Bioinformatics. 2023;24(1):63. 36823524 10.1186/s12859-023-05183-6 PMC9950014

[15] Luo Y, Kong Q, Yang J, Golden G, Wanda S-Y, Jensen RV, et al. Complete genome sequence of the Universal Killer *Salmonella enterica* Serovar Typhimurium UK-1 (ATCC 68169). J Bacteriol. 2011;193(15):4035–6. 21622747 10.1128/JB.05224-11 PMC3147501

[16] Luo Y, Kong Q, Yang J, Mitra A, Golden G, Wanda S-Y, et al. Comparative genome analysis of the high pathogenicity *Salmonella* Typhimurium Strain UK-1. PLoS ONE. 2012;7(7):e40645. 22792393 10.1371/journal.pone.0040645 PMC3391293

[17] Swaminathan B, Barrett TJ, Hunter SB, Tauxe RV. Pulsenet: the molecular subtyping network for foodborne bacterial disease surveillance. Emerg Infect Dis. 2001;7(3):382–9. 11384513 10.3201/eid0703.010303 PMC2631779

[18] Tolar B, Joseph LA, Schroeder MN, Stroika S, Ribot EM, Hise KB, et al. An overview of PulseNet USA databases. Foodborne Pathog Dis. 2019;16(7):457–62. 31066584 10.1089/fpd.2019.2637 PMC6653802

[19] Wickham H, Averick M, Bryan J, Chang W, McGowan LDA, François R, et al. Welcome to the tidyverse. J Open Source Softw. 2019;4(43):1686.

[20] R Core Team. R: A Language and Environment for Statistical Computing. Vienna, Austria: R Foundation for Statistical Computing. 2024. Available from: https://cran.rstudio.com/manuals.html. Accessed 2 July 2024

[21] RStudio Team. RStudio: Integrated Development Environment for R. Boston, MA. 2024.

[22] Grolemund G, Wickham H. Dates and times made easy with lubridate. J Stat Softw. 2011;40(3):1–25.

[23] Microsoft Corporation, Folashade D, Weston S. doParallel: Foreach Parallel Adaptor for the 'parallel' Package 2022. Available from: https://rdrr.io/cran/doParallel/. Accessed 30 Aug 2024

[24] Microsoft Corporation, Weston S. foreach: Provides Foreach Looping Construct 2022. Available from: https://rdrr.io/cran/foreach/man/foreach-package.html. Accessed 30 Aug 2024

[25] Pagès H, Aboyoun P, Gentleman R, DebRoy S. Biostrings: Efficient manipulation of biological strings 2024. Available from: https://bioconductor.org/packages/release/bioc/html/Biostrings.html. Accessed 30 Aug 2024

[26] Paradis E, Schliep K. Ape 5.0: an environment for modern phylogenetics and evolutionary analyses in R. Bioinformatics. 2019;35(3):526–8. 30016406 10.1093/bioinformatics/bty633

[27] Wickham H. The split-apply-combine strategy for data analysis. J Stat Softw. 2011;40(1):1–29.

[28] Wickham H, Pedersen TL, Seidel D. scales: Scale Functions for Visualization 2023. Available from: https://scales.r-lib.org/. Accessed 30 Aug 2024

[29] Osorio D, Rondón-Villarreal P, Torres R. Peptides: a package for data mining of antimicrobial peptides. R J. 2015;7(1):4–14.

[30] Dalsass M, Brozzi A, Medini D, Rappuoli R. Comparison of open-source reverse vaccinology programs for bacterial vaccine antigen discovery. Front Immunol. 2019;10:113. 30837982 10.3389/fimmu.2019.00113 PMC6382693

[31] Ong E, Cooke MF, Huffman A, Xiang Z, Wong MU, Wang H, et al. Vaxign2: The second generation of the first Web-based vaccine design program using reverse vaccinology and machine learning. Nucleic Acids Res. 2021;49(W1):W671–8. 34009334 10.1093/nar/gkab279 PMC8218197

[32] Ong E, Wang H, Wong MU, Seetharaman M, Valdez N, He Y. Vaxign-ML: supervised machine learning reverse vaccinology model for improved prediction of bacterial protective antigens. Bioinformatics. 2020;36(10):3185–91. 32096826 10.1093/bioinformatics/btaa119 PMC7214037

[33] Poudel S, Jia L, Arick MA II, Hsu C-Y, Thrash A, Sukumaran AT, et al. *In** silico* prediction and expression analysis of vaccine candidate genes of *Campylobacter* jejuni. Poult Sci. 2023;102(5):102592. 36972674 10.1016/j.psj.2023.102592 PMC10066559

[34] Yu NY, Wagner JR, Laird MR, Melli G, Rey S, Lo R, et al. PSORTb 3.0: improved protein subcellular localization prediction with refined localization subcategories and predictive capabilities for all prokaryotes. Bioinformatics. 2010;26(13):1608–15. 20472543 10.1093/bioinformatics/btq249 PMC2887053

[35] Ullah A, Ahmad S, Ismail S, Afsheen Z, Khurram M, Tahir ul Qamar M, et al. Towards A novel multi-epitopes chimeric vaccine for simulating strong immune responses and protection against *Morganella Morganii*. Int J Environ Res Public Health. 2021;18(20):10961. 34682706 10.3390/ijerph182010961 PMC8535705

[36] Ghosh S, Chakraborty K, Nagaraja T, Basak S, Koley H, Dutta S, et al. An adhesion protein of *Salmonella enterica* serovar Typhi is required for pathogenesis and potential target for vaccine development. Proc Natl Acad Sci. 2011;108(8):3348–53. 21300870 10.1073/pnas.1016180108 PMC3044360

[37] He Y, Xiang Z, Mobley HL. Vaxign: the first web-based vaccine design program for reverse vaccinology and applications for vaccine development. J Biomed Biotechnol. 2010;2010:297505. 20671958 10.1155/2010/297505 PMC2910479

[38] Sachdeva G, Kumar K, Jain P, Ramachandran S. SPAAN: a software program for prediction of adhesins and adhesin-like proteins using neural networks. Bioinformatics. 2005;21(4):483–91. 15374866 10.1093/bioinformatics/bti028 PMC7109999

[39] Krogh A, Larsson B, Von Heijne G, Sonnhammer EL. Predicting transmembrane protein topology with a hidden Markov model: application to complete genomes. J Mol Biol. 2001;305(3):567–80. 11152613 10.1006/jmbi.2000.4315

[40] Pizza M, Scarlato V, Masignani V, Giuliani MM, Arico B, Comanducci M, et al. Identification of vaccine candidates against serogroup b meningococcus by whole-genome sequencing. Science. 2000;287(5459):1816–20. 10710308 10.1126/science.287.5459.1816

[41] Jiang Z, Kang X, Song Y, Zhou X, Yue M. Identification and evaluation of novel antigen candidates against *Salmonella pullorum* infection using reverse vaccinology. Vaccines (Basel). 2023;11(4):865. 37112777 10.3390/vaccines11040865 PMC10143441

[42] Camacho C, Coulouris G, Avagyan V, Ma N, Papadopoulos J, Bealer K, et al. BLAST+: architecture and applications. BMC Bioinformatics. 2009;10:421. 20003500 10.1186/1471-2105-10-421 PMC2803857

[43] Sayers EW, Beck J, Bolton EE, Bourexis D, Brister JR, Canese K, et al. Database resources of the National Center for Biotechnology Information. Nucleic Acids Res. 2021;49(D1):D10-17. 33095870 10.1093/nar/gkaa892 PMC7778943

[44] Doytchinova IA, Flower DR. VaxiJen: a server for prediction of protective antigens, tumour antigens and subunit vaccines. BMC Bioinformatics. 2007;8:4. 17207271 10.1186/1471-2105-8-4 PMC1780059

[45] Gul I, Hassan A, Muneeb JM, Akram T, Haq E, Shah RA, et al. A multiepitope vaccine candidate against infectious bursal disease virus using immunoinformatics-based reverse vaccinology approach. Front Vet Sci. 2023;9:1116400. 36713875 10.3389/fvets.2022.1116400 PMC9880294

[46] Bhattacharjee A, Hosen MR, Lamisa AB, Ahammad I, Chowdhury ZM, Jamal TB, et al. An integrated comparative genomics, subtractive proteomics and immunoinformatics framework for the rational design of a Pan-*Salmonella* multi-epitope vaccine. PLoS ONE. 2024;19(7):e0292413. 38959229 10.1371/journal.pone.0292413 PMC11221655

[47] Valdivia-Olarte H, Requena D, Ramirez M, Saravia LE, Izquierdo R, Falconi-Agapito F, et al. Design of a predicted MHC restricted short peptide immunodiagnostic and vaccine candidate for Fowl adenovirus C in chicken infection. Bioinformation. 2015;11(10):460. 26664030 10.6026/97320630011460 PMC4658644

[48] Calis JJ, Maybeno M, Greenbaum JA, Weiskopf D, De Silva AD, Sette A, et al. Properties of MHC class I presented peptides that enhance immunogenicity. PLoS Comput Biol. 2013;9(10):e1003266. 24204222 10.1371/journal.pcbi.1003266 PMC3808449

[49] Gupta S, Kapoor P, Chaudhary K, Gautam A, Kumar R, Consortium OSDD, et al. In silico approach for predicting toxicity of peptides and proteins. PLoS One. 2013;8(9):e73957. 24058508 10.1371/journal.pone.0073957 PMC3772798

[50] Kyte J, Doolittle RF. A simple method for displaying the hydropathic character of a protein. J Mol Biol. 1982;157(1):105–32. 7108955 10.1016/0022-2836(82)90515-0

[51] Kans J. Entrez direct: E-utilities on the UNIX command line. Entrez programming utilities help: National Center for Biotechnology Information (US); 2024. Available from: https://www.ncbi.nlm.nih.gov/books/NBK179288/.

[52] Kringelum JV, Nielsen M, Padkjær SB, Lund O. Structural analysis of B-cell epitopes in antibody: protein complexes. Mol Immunol. 2013;53(1–2):24–34. 22784991 10.1016/j.molimm.2012.06.001 PMC3461403

[53] Ras-Carmona A, Lehmann AA, Lehmann PV, Reche PA. Prediction of B cell epitopes in proteins using a novel sequence similarity-based method. Sci Rep. 2022;12(1):13739. 35962028 10.1038/s41598-022-18021-1 PMC9374694

[54] Gloanec N, Guyard-Nicodème M, Chemaly M, Dory D. Reverse vaccinology: a strategy also used for identifying potential vaccine antigens in poultry. Vaccine. 2025;48:126756. 39855107 10.1016/j.vaccine.2025.126756

[55] Vij S, Thakur R, Rishi P. Reverse engineering approach: a step towards a new era of vaccinology with special reference to *Salmonella*. Expert Rev Vaccines. 2022;21(12):1763–85. 36408592 10.1080/14760584.2022.2148661

[56] Mugunthan SP, Mani CH. A computational reverse vaccinology approach for the design and development of multi-epitopic vaccine against avian pathogen *Mycoplasma Gallisepticum*. Front Vet Sci. 2021;8:721061. 34765664 10.3389/fvets.2021.721061 PMC8577832

[57] Fatoba AJ, Adeleke VT, Maharaj L, Okpeku M, Adeniyi AA, Adeleke MA. Design of a multiepitope vaccine against chicken anemia virus disease. Viruses. 2022;14(7):1456. 35891436 10.3390/v14071456 PMC9318905

[58] Raza A, Rasheed MA, Raza S, Navid MT, Afzal A, Jamil F. Prediction and analysis of multi epitope based vaccine against Newcastle disease virus based on haemagglutinin neuraminidase protein. Saudi J Biol Sci. 2022;29(4):3006–14. 35531218 10.1016/j.sjbs.2022.01.036 PMC9073007

[59] Nezafat N, Ghasemi Y, Javadi G, Khoshnoud MJ, Omidinia E. A novel multi-epitope peptide vaccine against cancer: an *in silico* approach. J Theor Biol. 2014;349:121–34. 24512916 10.1016/j.jtbi.2014.01.018

[60] Kolla HB, Tirumalasetty C, Sreerama K, Ayyagari VS. An immunoinformatics approach for the design of a multi-epitope vaccine targeting super antigen TSST-1 of *Staphylococcus aureus*. J Genet Eng Biotechnol. 2021;19(1):69. 33974183 10.1186/s43141-021-00160-z PMC8112219

[61] Rahmani A, Baee M, Rostamtabar M, Karkhah A, Alizadeh S, Tourani M, et al. Development of a conserved chimeric vaccine based on helper T-cell and CTL epitopes for induction of strong immune response against Schistosoma mansoni using immunoinformatics approaches. Int J Biol Macromol. 2019;141:125–36. 31479669 10.1016/j.ijbiomac.2019.08.259

[62] Dimitrov I, Naneva L, Doytchinova I, Bangov I. AllergenFP: allergenicity prediction by descriptor fingerprints. Bioinformatics. 2014;30(6):846–51. 24167156 10.1093/bioinformatics/btt619

[63] Magnan CN, Randall A, Baldi P. SOLpro: accurate sequence-based prediction of protein solubility. Bioinformatics. 2009;25(17):2200–7. 19549632 10.1093/bioinformatics/btp386

[64] Cheng J, Randall AZ, Sweredoski MJ, Baldi P. SCRATCH: a protein structure and structural feature prediction server. Nucleic Acids Res. 2005;33(Suppl_2):W72–6. 15980571 10.1093/nar/gki396 PMC1160157

[65] Mahapatra SR, Dey J, Kushwaha GS, Puhan P, Mohakud NK, Panda SK, et al. Immunoinformatic approach employing modeling and simulation to design a novel vaccine construct targeting MDR efflux pumps to confer wide protection against typhoidal *Salmonella* serovars. J Biomol Struct Dyn. 2022;40(22):11809–21. 34463211 10.1080/07391102.2021.1964600

[66] Sharma N, Naorem LD, Jain S, Raghava GP. ToxinPred2: an improved method for predicting toxicity of proteins. Brief Bioinform. 2022;23(5):bbac174. 35595541 10.1093/bib/bbac174

[67] Gasteiger E, Hoogland C, Gattiker A, Duvaud Se, Wilkins MR, Appel RD, et al. Protein identification and analysis tools on the ExPASy server. In: Walker, J.M. (eds) The Proteomics Protocols Handbook. Springer Protocols Handbooks. Humana Press.; 2005. 531–552.

[68] Guruprasad K, Reddy BB, Pandit MW. Correlation between stability of a protein and its dipeptide composition: a novel approach for predicting *in vivo* stability of a protein from its primary sequence. Protein Eng. 1990;4(2):155–61. 2075190 10.1093/protein/4.2.155

[69] Ikai A. Thermostability and aliphatic index of globular proteins. J Biochem. 1980;88(6):1895–8. 7462208

[70] Buchan DWA, Jones DT. The PSIPRED protein analysis workbench: 20 years on. Nucleic Acids Res. 2019;47(W1):W402–7. 31251384 10.1093/nar/gkz297 PMC6602445

[71] Jones DT. Protein secondary structure prediction based on position-specific scoring matrices. J Mol Biol. 1999;292(2):195–202. 10493868 10.1006/jmbi.1999.3091

[72] Kelley LA, Mezulis S, Yates CM, Wass MN, Sternberg MJE. The Phyre2 web portal for protein modeling, prediction and analysis. Nat Protoc. 2015;10(6):845–58. 25950237 10.1038/nprot.2015.053 PMC5298202

[73] Meng EC, Goddard TD, Pettersen EF, Couch GS, Pearson ZJ, Morris JH, et al. UCSF ChimeraX: tools for structure building and analysis. Protein Sci. 2023;32(11):e4792. 37774136 10.1002/pro.4792 PMC10588335

[74] Heo L, Park H, Seok C. GalaxyRefine: protein structure refinement driven by side-chain repacking. Nucleic Acids Res. 2013;41(W1):W384-8. 23737448 10.1093/nar/gkt458 PMC3692086

[75] Modi V, Dunbrack RL Jr. Assessment of refinement of template-based models in CASP11. Proteins. 2016;84:260–81. 27081793 10.1002/prot.25048 PMC5030126

[76] Chen VB, Arendall WB 3rd, Headd JJ, Keedy DA, Immormino RM, Kapral GJ, et al. MolProbity: all-atom structure validation for macromolecular crystallography. Acta Crystallogr D Biol Crystallogr. 2010;66(Pt 1):12–21. 20057044 10.1107/S0907444909042073 PMC2803126

[77] Eisenberg D, Lüthy R, Bowie JU. VERIFY3D: assessment of protein models with three-dimensional profiles. Methods Enzymol. 1997;277:396–404. 9379925 10.1016/s0076-6879(97)77022-8

[78] Colovos C, Yeates TO. Verification of protein structures: patterns of nonbonded atomic interactions. Protein Sci. 1993;2(9):1511–9. 8401235 10.1002/pro.5560020916 PMC2142462

[79] Isaacs D, Mikasi SG, Obasa AE, Ikomey GM, Shityakov S, Cloete R, et al. Structural Comparison of Diverse HIV-1 Subtypes using Molecular Modelling and Docking Analyses of Integrase Inhibitors. Viruses. 2020;12(9):936. 32858802 10.3390/v12090936 PMC7552036

[80] Laskowski RA, MacArthur MW, Moss DS, Thornton JM. PROCHECK: a program to check the stereochemical quality of protein structures. J Appl Cryst. 1993;26(2):283–91.

[81] Rossmann MG, Arnold E. International Tables for Crystallography Volume F: Crystallography of biological macromolecules. Wiley Online Library; 2001.

[82] Wiederstein M, Sippl MJ. ProSA-web: interactive web service for the recognition of errors in three-dimensional structures of proteins. Nucleic Acids Res. 2007;35(Suppl_2):W407-10. 17517781 10.1093/nar/gkm290 PMC1933241

[83] Neerukonda SN, Katneni U. Avian pattern recognition receptor sensing and signaling. Vet Sci. 2020;7(1):14. 32012730 10.3390/vetsci7010014 PMC7157566

[84] Abadi MHJN, Abyaneh FA, Zare N, Zamani J, Abdoli A, Aslanbeigi F, et al. *In silico* design and immunoinformatics analysis of a chimeric vaccine construct based on *Salmonella* pathogenesis factors. Microb Pathog. 2023;180:106130. 37121524 10.1016/j.micpath.2023.106130

[85] Zafar S, Ajab H, Baig S, Baig A, Habib Z, Jamil F, et al. Prediction and evaluation of multi epitope based sub-unit vaccine against *Salmonella* typhimurium. Saudi J Biol Sci. 2022;29(2):1092–9. 35197778 10.1016/j.sjbs.2021.09.061 PMC8847936

[86] Jin MS, Kim SE, Heo JY, Lee ME, Kim HM, Paik S-G, et al. Crystal structure of the TLR1-TLR2 heterodimer induced by binding of a tri-acylated lipopeptide. Cell. 2007;130(6):1071–82. 17889651 10.1016/j.cell.2007.09.008

[87] Manavalan B, Basith S, Choi S. Similar structures but different roles–an updated perspective on TLR structures. Front Physiol. 2011;2:41. 21845181 10.3389/fphys.2011.00041 PMC3146039

[88] Kozakov D, Hall DR, Xia B, Porter KA, Padhorny D, Yueh C, et al. The ClusPro web server for protein–protein docking. Nat Protoc. 2017;12(2):255–78. 28079879 10.1038/nprot.2016.169 PMC5540229

[89] Desta IT, Porter KA, Xia B, Kozakov D, Vajda S. Performance and its limits in rigid body protein-protein docking. Structure. 2020;28(9):1071-81.e3. 32649857 10.1016/j.str.2020.06.006 PMC7484347

[90] Kozakov D, Beglov D, Bohnuud T, Mottarella SE, Xia B, Hall DR, et al. How good is automated protein docking? Proteins. 2013;81(12):2159–66. 23996272 10.1002/prot.24403 PMC3934018

[91] Vajda S, Yueh C, Beglov D, Bohnuud T, Mottarella SE, Xia B, et al. New additions to the Clus Pro server motivated by CAPRI. Proteins. 2017;85(3):435–44. 27936493 10.1002/prot.25219 PMC5313348

[92] Laskowski RA, Jabłońska J, Pravda L, Vařeková RS, Thornton JM. PDBsum: structural summaries of PDB entries. Protein Sci. 2018;27(1):129–34. 28875543 10.1002/pro.3289 PMC5734310

[93] Abraham MJ, Murtola T, Schulz R, Páll S, Smith JC, Hess B, et al. GROMACS: High performance molecular simulations through multi-level parallelism from laptops to supercomputers. SoftwareX. 2015;1–2:19–25.

[94] Pronk S, Páll S, Schulz R, Larsson P, Bjelkmar P, Apostolov R, et al. GROMACS 4.5: a high-throughput and highly parallel open source molecular simulation toolkit. Bioinformatics. 2013;29(7):845–54. 23407358 10.1093/bioinformatics/btt055 PMC3605599

[95] Abraham M, Alekseenko A, Bergh C, Blau C, Briand Eliane, Doijade M, et al. GROMACS 2023.3 Manual 2023 Available from: https://zenodo.org/records/10017699. Access13 Dec 2023

[96] CompChems. GROMACS Tutorial: Molecular Dynamics simulation of a protein in water environment 2023 Available from: https://www.compchems.com/gromacs-tutorial-molecular-dynamics-simulation-of-a-protein-in-water-environment/.

[97] Dimitrov D. GROMACS Tutorial. A Guide to Molecular Dynamics Using GROMACS. Available from: https://static.igem.org/mediawiki/2021/d/d2/T--KCL_UK--UPDATEDGROMACSGUIDE.pdf.

[98] Alves AC, Magarkar A, Horta M, Lima JLFC, Bunker A, Nunes C, et al. Influence of doxorubicin on model cell membrane properties: insights from *in vitro* and *in silico* studies. Sci Rep. 2017;7(1):6343. 28740256 10.1038/s41598-017-06445-z PMC5524714

[99] Cândido MGL, Tinôco IFF, Albino LFT, Freitas LCSR, Santos TC, Cecon PR, et al. Effects of heat stress on pullet cloacal and body temperature. Poult Sci. 2020;99(5):2469–77. 32359582 10.1016/j.psj.2019.11.062 PMC7597385

[100] Khan RU, Naz S, Ullah H, Ullah Q, Laudadio V, Qudratullah, et al. Physiological dynamics in broiler chickens under heat stress and possible mitigation strategies. Anim Biotechnol. 2023;34(2):438–47. 34473603 10.1080/10495398.2021.1972005

[101] Bemani P, Amirghofran Z, Mohammadi M. Designing a multi-epitope vaccine against blood-stage of *Plasmodium falciparum* by in silico approaches. J Mol Graph Model. 2020;99:107645. 32454399 10.1016/j.jmgm.2020.107645

[102] Saleki K, Mohamadi MH, Banazadeh M, Alijanizadeh P, Javanmehr N, Pourahmad R, et al. *In silico* design of a TLR4-mediating multiepitope chimeric vaccine against amyotrophic lateral sclerosis via advanced immunoinformatics. J Leukoc Biol. 2022;112(5):1191–207. 35707959 10.1002/JLB.6MA0721-376RR

[103] Umitaibatin R, Harisna AH, Jauhar MM, Syaifie PH, Arda AG, Nugroho DW, et al. Immunoinformatics study: multi-epitope based vaccine design from SARS-CoV-2 Spike glycoprotein. Vaccines (Basel). 2023;11(2):399. 36851275 10.3390/vaccines11020399 PMC9964839

[104] Berendsen HJ, Grigera JR, Straatsma TP. The missing term in effective pair potentials. J Phys Chem. 1987;91(24):6269–71.

[105] Kaminski GA, Friesner RA, Tirado-Rives J, Jorgensen WL. Evaluation and reparametrization of the OPLS-AA force field for proteins via comparison with accurate quantum chemical calculations on peptides. J Phys Chem B. 2001;105(28):6474–87.

[106] Rabienia M, Roudbari Z, Ghanbariasad A, Abdollahi A, Mohammadi E, Mortazavidehkordi N, et al. Exploring membrane proteins of Leishmania major to design a new multi-epitope vaccine using immunoinformatics approach. Eur J Pharm Sci. 2020;152:105423. 32534195 10.1016/j.ejps.2020.105423

[107] Castiglione F, Bernaschi M. C-ImmSim: playing with the immune response. In Proceedings of the sixteenth International symposium on mathematical theory of networks and systems (MTNS2004). Katholieke Universiteit Leuven Belgium. 2004:1–7.

[108] Ragone C, Manolio C, Cavalluzzo B, Mauriello A, Tornesello ML, Buonaguro FM, et al. Identification and validation of viral antigens sharing sequence and structural homology with tumor-associated antigens (TAAs). J ImmunoTher Cancer. 2021;9(5):e002694. 34049932 10.1136/jitc-2021-002694 PMC8166618

[109] Rapin N, Lund O, Bernaschi M, Castiglione F. Computational immunology meets bioinformatics: the use of prediction tools for molecular binding in the simulation of the immune system. PLoS ONE. 2010;5(4):e9862. 20419125 10.1371/journal.pone.0009862 PMC2855701

[110] Stolfi P, Castiglione F, Mastrostefano E, Di Biase I, Di Biase S, Palmieri G, et al. In-silico evaluation of adenoviral COVID-19 vaccination protocols: assessment of immunological memory up to 6 months after the third dose. Front immunol. 2022;13:998262. 36353634 10.3389/fimmu.2022.998262 PMC9639861

[111] Chen S, Zhou Y, Chen Y, Gu J. fastp: an ultra-fast all-in-one FASTQ preprocessor. Bioinformatics. 2018;34(17):i884–90. 30423086 10.1093/bioinformatics/bty560 PMC6129281

[112] Souvorov A, Agarwala R, Lipman DJ. SKESA: strategic k-mer extension for scrupulous assemblies. Genome Biol. 2018;19(1):153. 30286803 10.1186/s13059-018-1540-z PMC6172800

[113] Parks DH, Imelfort M, Skennerton CT, Hugenholtz P, Tyson GW. CheckM: assessing the quality of microbial genomes recovered from isolates, single cells, and metagenomes. Genome Res. 2015;25(7):1043–55. 25977477 10.1101/gr.186072.114 PMC4484387

[114] Ismail S, Ahmad S, Azam SS. Vaccinomics to design a novel single chimeric subunit vaccine for broad-spectrum immunological applications targeting nosocomial Enterobacteriaceae pathogens. Eur J Pharm Sci. 2020;146:105258. 32035109 10.1016/j.ejps.2020.105258

[115] Tabassum R, Abbas G, Azam SS. Immunoinformatics based designing and simulation of multi-epitope vaccine against multi-drug resistant *Stenotrophomonas maltophilia*. J Mol Liq. 2021;340:116899.

[116] Li J, Qiu J, Huang Z, Liu T, Pan J, Zhang Q, et al. Reverse vaccinology approach for the identifications of potential vaccine candidates against *Salmonella*. Int J Med Microbiol. 2021;311(5):151508. 34182206 10.1016/j.ijmm.2021.151508

[117] Sirajee AS, Ahsan S. Design of a cross-protective multi-epitope vaccine targeting the most prevalent typhoidal and non-typhoidal *Salmonella* serovars. Heliyon. 2025;11(5):e42954.

[118] Priyadarsini S, Panda S, Pashupathi M, Kumar A, Singh R. Design of multiepitope vaccine construct against non-typhoidal salmonellosis and its characterization using immunoinformatics approach. Inte J Pept Res Ther. 2021;27:2333–48.

[119] Khan K, Jalal K, Uddin R. An integrated in silico based subtractive genomics and reverse vaccinology approach for the identification of novel vaccine candidate and chimeric vaccine against XDR *Salmonella typhi* H58. Genomics. 2022;114(2):110301. 35149170 10.1016/j.ygeno.2022.110301

[120] Esmailnia E, Amani J, Gargari SLM. Identification of novel vaccine candidate against *Salmonella enterica* serovar Typhi by reverse vaccinology method and evaluation of its immunization. Genomics. 2020;112(5):3374–81. 32565239 10.1016/j.ygeno.2020.06.022

[121] Li Q, Ren J, Xian H, Yin C, Yuan Y, Li Y, et al. rOmpF and OMVs as efficient subunit vaccines against *Salmonella enterica* serovar Enteritidis infections in poultry farms. Vaccine. 2020;38(45):7094–9. 32951940 10.1016/j.vaccine.2020.08.074

[122] Luo Y, Zeng Q, Glisson JR, Jackwood MW, Cheng IHN, Wang C. Sequence analysis of *Pasteurella multocida* major outer membrane protein (OmpH) and application of synthetic peptides in vaccination of chickens against homologous strain challenge. Vaccine. 1999;17(7–8):821–31. 10067687 10.1016/s0264-410x(98)00266-7

[123] Humphries A, DeRidder S, Bäumler AJ. *Salmonella enterica* serotype Typhimurium fimbrial proteins serve as antigens during infection of mice. Infect Immun. 2005;73(9):5329–38. 16113248 10.1128/IAI.73.9.5329-5338.2005 PMC1231134

[124] Su GF, Brahmbhatt H, Wehland J, Rohde M, Timmis K. Construction of stable LamB-Shiga toxin B subunit hybrids: analysis of expression in *Salmonella typhimurium* aroA strains and stimulation of B subunit-specific mucosal and serum antibody responses. Infect Immun. 1992;60(8):3345–59. 1639503 10.1128/iai.60.8.3345-3359.1992 PMC257321

[125] Yang T-C, Ma X-C, Liu F, Lin L-R, Liu L-L, Liu G-L, et al. Screening of the *Salmonella* paratyphi A CMCC 50973 strain outer membrane proteins for the identification of potential vaccine targets. Molecular Med Rep. 2012;5(1):78–83. 10.3892/mmr.2011.587 21922141

[126] Yang Y, Wan C, Xu H, Wei H. Identification and characterization of OmpL as a potential vaccine candidate for immune-protection against salmonellosis in mice. Vaccine. 2013;31(28):2930–6. 23643894 10.1016/j.vaccine.2013.04.044

[127] Toobak H, Rasooli I, Talei D, Jahangiri A, Owlia P, Astaneh SDA. Immune response variations to *Salmonella enterica* serovar Typhi recombinant porin proteins in mice. Biologicals. 2013;41(4):224–30. 23796754 10.1016/j.biologicals.2013.05.005

[128] Lun J, Xia C, Yuan C, Zhang Y, Zhong M, Huang T, et al. The outer membrane protein, LamB (maltoporin), is a versatile vaccine candidate among the Vibrio species. Vaccine. 2014;32(7):809–15. 24380680 10.1016/j.vaccine.2013.12.035

[129] Rowley G, Skovierova H, Stevenson A, Rezuchova B, Homerova D, Lewis C, et al. The periplasmic chaperone Skp is required for successful *Salmonella* Typhimurium infection in a murine typhoid model. Microbiology. 2011;157(3):848–58. 21148205 10.1099/mic.0.046011-0

[130] Kong Q, Six DA, Liu Q, Gu L, Roland KL, Raetz CR, et al. Palmitoylation state impacts induction of innate and acquired immunity by the *Salmonella enterica* serovar Typhimurium msbB mutant. Infect immun. 2011;79(12):5027–38. 21930761 10.1128/IAI.05524-11 PMC3232669

[131] Kong Q, Six DA, Liu Q, Gu L, Wang S, Alamuri P, et al. Phosphate groups of lipid A are essential for *Salmonella enterica* serovar Typhimurium virulence and affect innate and adaptive immunity. Infect immun. 2012;80(9):3215–24. 22753374 10.1128/IAI.00123-12 PMC3418755

[132] Zhang HP, Kang YH, Kong LC, Ju AQ, Wang YM, Muhammad I, et al. Functional analysis of hisJ in *Aeromonas veronii* reveals a key role in virulence. Ann N Y Acad Sci. 2020;1465(1):146–60. 31663616 10.1111/nyas.14265

[133] Zhu C, Xiong K, Chen Z, Hu X, Li J, Wang Y, et al. Construction of an attenuated *Salmonella enterica* serovar Paratyphi A vaccine strain harboring defined mutations in htrA and yncD. Microbiol immun. 2015;59(8):443–51. 10.1111/1348-0421.12276 26084199

[134] Morris FC, Wells TJ, Bryant JA, Schager AE, Sevastsyanovich YR, Squire DJ, et al. YraP contributes to cell envelope integrity and virulence of *Salmonella enterica* serovar Typhimurium. Infect immun. 2018;86(11):10–128. 10.1128/iai.00829-17. PMC6204713 30201701

[135] Xiong K, Chen Z, Xiang G, Wang J, Rao X, Hu F, et al. Deletion of yncD gene in Salmonella enterica ssp. enterica serovar Typhi leads to attenuation in mouse model. FEMS Microbiol Lett. 2012;328(1):70–7. 22150228 10.1111/j.1574-6968.2011.02481.x

[136] Chatfield SN, Dorman CJ, Hayward C, Dougan G. Role of *ompR*-dependent genes in *Salmonella typhimurium* virulence: mutants deficient in both *ompC* and *ompF* are attenuated in vivo. Infect Immun. 1991;59(1):449–52. 1846127 10.1128/iai.59.1.449-452.1991 PMC257763

[137] Hermans D, Pasmans F, Heyndrickx M, Van Immerseel F, Martel A, Van Deun K, et al. A tolerogenic mucosal immune response leads to persistent *Campylobacter jejuni* colonization in the chicken gut. Crit Rev Microbiol. 2012;38(1):17–29. 21995731 10.3109/1040841X.2011.615298

[138] Härtle S, Magor KE, Göbel TW, Davison F, Kaspers B. Chapter 5 - Structure and evolution of avian immunoglobulins. In: Kaspers B, Schat KA, Göbel TW, Vervelde L, editors. Avian Immunology. 3rd ed. Boston: Academic Press; 2022. p. 101–19.

[139] Kogut MH. Cytokines and prevention of infectious diseases in poultry: a review. Avian Pathol. 2000;29(5):395–404. 19184830 10.1080/030794500750047135

[140] von La RD, Schumacher M, Kohn M, Trapp J, Schusser B, Rautenschlein S, et al. Characterization of class-switched B cells in chickens. Front Immunol. 2024;15:1484288. 39640270 10.3389/fimmu.2024.1484288 PMC11617357

[141] Leeper MM, Tolar BM, Griswold T, Vidyaprakash E, Hise KB, Williams GM, et al. Evaluation of whole and core genome multilocus sequence typing allele schemes for *Salmonella enterica* outbreak detection in a national surveillance network. PulseNet USA Front Microbiol. 2023;14:1254777. 37808298 10.3389/fmicb.2023.1254777 PMC10558246

[142] Verma S, Sugadev R, Kumar A, Chandna S, Ganju L, Bansal A. Multi-epitope DnaK peptide vaccine against *S. Typhi*: an in silico approach. Vaccine. 2018;36(28):4014–22. 29861180 10.1016/j.vaccine.2018.05.106

[143] Ali MC, Khatun MS, Jahan SI, Das R, Munni YA, Rahman MM, et al. *In silico* design of epitope-based peptide vaccine against non-typhoidal *Salmonella* through immunoinformatic approaches. J Biomolecular Struct Dyn. 2022;40(21):10696–714. 10.1080/07391102.2021.1947381 36529187

[144] Yeh H-Y. Epitope mapping of recombinant *Salmonella enterica* serotype Heidelberg flagellar hook-associated protein by in silico and in vivo approaches. BMC Vet Res. 2025;21(1):54. 39915877 10.1186/s12917-025-04479-4 PMC11803983

[145] Lv P, Zhang X, Song M, Hao G, Wang F, Sun S. Oral administration of recombinant *Bacillus subtilis* expressing a multi-epitope protein induces strong immune responses against *Salmonella* Enteritidis. Vet Microbiol. 2023;276:109632. 36521295 10.1016/j.vetmic.2022.109632

[146] Meniaï I, Thibodeau A, Quessy S, Parreira VR, Fravalo P, Beauchamp G, et al. Putative antigenic proteins identified by comparative and subtractive reverse vaccinology in necrotic enteritis-causing Clostridium perfringens isolated from broiler chickens. BMC Genomics. 2021;22(1):890. 34903179 10.1186/s12864-021-08216-7 PMC8666345

[147] Meunier M, Guyard-Nicodème M, Hirchaud E, Parra A, Chemaly M, Dory D. Identification of novel vaccine candidates against *Campylobacter* through reverse vaccinology. J Immunol Res. 2016;2016(1):5715790. 27413761 10.1155/2016/5715790 PMC4928009

[148] Al-Hasani K, Boyce J, McCarl VP, Bottomley S, Wilkie I, Adler B. Identification of novel immunogens in *Pasteurella multocida*. Microb Cell Fact. 2007;6(1):3. 17233917 10.1186/1475-2859-6-3 PMC1781955

[149] Ali SA, Almofti YA, Abd-Elrahman KA. Immunoinformatics approach for multiepitopes vaccine prediction against glycoprotein B of avian infectious laryngotracheitis virus. Adv Bioinformatics. 2019;2019(1):1270485. 31011331 10.1155/2019/1270485 PMC6442309

[150] Aziz F, Tufail S, Shah MA, Shah MS, Habib M, Mirza O, et al. *In silico* epitope prediction and immunogenic analysis for penton base epitope-focused vaccine against hydropericardium syndrome in chicken. Virus Res. 2019;273:197750. 31509776 10.1016/j.virusres.2019.197750

[151] Igomu EE, Mamman PH, Adamu J, Muhammad M, Woziri AO, Sugun MY, et al. Immunoinformatics design of a novel multiepitope vaccine candidate against non-typhoidal salmonellosis caused by *Salmonella* Kentucky using outer membrane proteins A, C, and F. PLoS ONE. 2025;20(1):e0306200. 39792829 10.1371/journal.pone.0306200 PMC11723559

[152] Sharma S, Solanki V, Tiwari V. Reverse vaccinology approach to design a vaccine targeting membrane lipoproteins of *Salmonella typhi*. J Biomol Struct Dyn. 2023;41(3):954–69. 34939517 10.1080/07391102.2021.2015443

[153] Smith J, Alfieri JM, Anthony N, Arensburger P, Athrey GN, Balacco J, et al. Fourth report on chicken genes and chromosomes 2022. Cytogenet Genome Res. 2023;162(8–9):405–528. 10.1159/000529376 PMC11835228 36716736

[154] Koch M, Camp S, Collen T, Avila D, Salomonsen J, Wallny H-J, et al. Structures of an MHC Class I Molecule from B21 chickens illustrate promiscuous peptide binding. Immunity. 2007;27(6):885–99. 18083574 10.1016/j.immuni.2007.11.007

[155] Xiao J, Xiang W, Zhang Y, Peng W, Zhao M, Niu L, et al. An invariant arginine in common with MHC Class II allows extension at the C-terminal end of peptides bound to chicken MHC Class I. J Immunol. 2018;201(10):3084–95. 30341185 10.4049/jimmunol.1800611

[156] Khan K, Samiullah B, Amer AA, Alhuthali HM, Alharthi NS, Jalal K. A therapeutic epitopes-based vaccine engineering against *Salmonella enterica* XDR strains for typhoid fever: a Pan-vaccinomics approach. J Biomol Struct Dyn. 2024;42(16):8559–73. 37578072 10.1080/07391102.2023.2246587

[157] Cao H, Xu H, Ning C, Xiang L, Ren Q, Zhang T, et al. Multi-omics approach reveals the potential core vaccine targets for the emerging foodborne pathogen *Campylobacter jejuni*. Front Microbiol. 2021;12:665858. 34248875 10.3389/fmicb.2021.665858 PMC8265506

[158] Mallaby J, Ng J, Stewart A, Sinclair E, Dunn-Walters D, Hershberg U. Chickens, more than humans, focus the diversity of their immunoglobulin genes on the complementarity-determining region but utilise amino acids, indicative of a more cross-reactive antibody repertoire. Front Immunol. 2022;13:837246. 36569888 10.3389/fimmu.2022.837246 PMC9772431

[159] Pérez-Toledo M, Valero-Pacheco N, Pastelin-Palacios R, Gil-Cruz C, Perez-Shibayama C, Moreno-Eutimio MA, et al. *Salmonella* Typhi porins OmpC and OmpF are potent adjuvants for T-dependent and T-independent antigens. Front immunol. 2017;8:230. 28337196 10.3389/fimmu.2017.00230 PMC5344031

[160] Secundino I, López-Macías C, Cervantes-Barragán L, Gil-Cruz C, Ríos-Sarabia N, Pastelin-Palacios R, et al. *Salmonella* porins induce a sustained, lifelong specific bactericidal antibody memory response. Immunology. 2006;117(1):59–70. 16423041 10.1111/j.1365-2567.2005.02263.x PMC1782194

[161] Hejair HM, Zhu Y, Ma J, Zhang Y, Pan Z, Zhang W, et al. Functional role of ompF and ompC porins in pathogenesis of avian pathogenic *Escherichia coli*. Microb pathog. 2017;107:29–37. 28315387 10.1016/j.micpath.2017.02.033

[162] Ghadi FE, Roudbari Z, Razavi R. Multi-epitope Peptide Vaccine Designing Based on OmpF, OmpC, and PgtE of *Salmonella enterica* Typhi by Computational Approaches. https://www.authorea.com/users/575068/articles/618489-multi-epitope-peptide-vaccine-designing-based-on-ompf-ompc-and-pgte-of-salmonella-enterica-typhi-by-computational-approaches?commit=dd71ce6ee5ea0ef4e91d19a9ca45c9dc23089b54 2023. Accessed 14 Aug 2023

[163] Gil-Marqués ML, Labrador Herrera G, Miró Canturri A, Pachón J, Smani Y, Pachón-Ibáñez ME. Role of PstS in the pathogenesis of *Acinetobacter baumannii* under microaerobiosis and normoxia. J Infect Dis. 2020;222(7):1204–12. 32324853 10.1093/infdis/jiaa201

[164] Wang J, Xiong K, Pan Q, He WF, Cong YG. Application of TonB-dependent transporters in vaccine development of Gram-negative bacteria. Front Cell Infect Microbiol. 2021;10:589115. 33585268 10.3389/fcimb.2020.589115 PMC7873555

[165] Kaneshige T, Yaguchi K, Ohgitani T. Siderophore receptor iron is an important protective antigen against *Salmonella* infection in chickens. Avian Dis. 2009;53(4):563–7. 20095157 10.1637/8925-051309-Reg.1

[166] Wieser A, Romann E, Magistro G, Hoffmann C, Nörenberg D, Weinert K, et al. A multiepitope subunit vaccine conveys protection against extraintestinal pathogenic *Escherichia coli* in mice. Infect immun. 2010;78(8):3432–42. 20498257 10.1128/IAI.00174-10 PMC2916271

[167] Siddique A, Wang Z, Zhou H, Huang L, Jia C, Wang B, et al. The evolution of vaccines development across *Salmonella* serovars among animal hosts: a systematic review. Vaccines (Basel). 2024;12(9):1067. 39340097 10.3390/vaccines12091067 PMC11435802

[168] Edrington TS, Arthur TM, Loneragan GH, Genovese KJ, Hanson DL, Anderson RC, et al. Evaluation of two commercially-available *Salmonella* vaccines on *Salmonella* in the peripheral lymph nodes of experimentally-infected cattle. Ther Adv Vaccines and Immunother. 2020;8:2515135520957760. 33089062 10.1177/2515135520957760 PMC7543105

[169] Nuccio SP, Baumler AJ. Evolution of the chaperone/usher assembly pathway: fimbrial classification goes Greek. Microbiol Mol Biol Rev. 2007;71(4):551–75. 18063717 10.1128/MMBR.00014-07 PMC2168650

[170] El-Maghraby AS, Mwafy A, El-Sawy H. Sequencing of *bcfC* gene of *Salmonella* Typhimurium isolated from ducks in Egypt. World Vet J. 2020;10(3):380–90.

[171] Elkenany R, Elsayed MM, Zakaria AI, El-Sayed SA, Rizk MA. Antimicrobial resistance profiles and virulence genotyping of *Salmonella enterica* serovars recovered from broiler chickens and chicken carcasses in Egypt. BMC Vet Res. 2019;15(1):124. 31029108 10.1186/s12917-019-1867-z PMC6486964

[172] Bishop RE. The lipid A palmitoyltransferase PagP: molecular mechanisms and role in bacterial pathogenesis. Mol Microbiol. 2005;57(4):900–12. 16091033 10.1111/j.1365-2958.2005.04711.x

[173] Guo L, Lim KB, Poduje CM, Daniel M, Gunn JS, Hackett M, et al. Lipid A acylation and bacterial resistance against vertebrate antimicrobial peptides. Cell. 1998;95(2):189–98. 9790526 10.1016/s0092-8674(00)81750-x

[174] Hantke K, Friz S. The TonB-dependent uptake of pyrroloquinoline-quinone (PQQ) and secretion of gluconate by *Escherichia coli* K-12. Mol Microbiol. 2022;118(4):417–25. 36054785 10.1111/mmi.14975

[175] Alonso A, Pucciarelli MG, Figueroa-Bossi N, García-del PF. Increased excision of the *Salmonella* prophage ST64B caused by a deficiency in Dam methylase. J Bacteriol. 2005;187(23):7901. 16291663 10.1128/JB.187.23.7901-7911.2005 PMC1291290

