# Supplementary material for: In silico design and evaluation of a candidate multiepitope Salmonella vaccine construct targeting broad-spectrum protection in poultry
Source: BMC Genomics. 2026 Jun 25;27:758. doi: 10.1186/s12864-026-13056-4 (PMC13584361; doi:10.1186/s12864-026-13056-4)
Supplement: Supplementary file 1 [file 12864_2026_13056_MOESM1_ESM.docx]

Phase I – Subtractive Proteomics Analysis

Vaxign2 (<https://violinet.org/vaxign2>)

The Vaxign-ML algorithm, which is used to predict protein sequence antigenicity, was trained on the biological and physicochemical properties of proteins found in the Protegen database which has ten years of experimentally verified protective antigens from published literature [1, 2]. Proteins from the *S.* Typhimurium str. UK-1 proteins faa file (n=4,555) were input into Vaxign2 in batches of 250 to not overwhelm the server. Proteins are analyzed on an individual basis, thus there are no batch effects.

PSORTb (Bacterial Protein Subcellular Localization Prediction Tool) (v3.0) predicts a proteins localization in the cell by looking for alpha helices with HMMTOP (Hidden Markov Model for Topology Prediction) and creates a generalized suffix tree to extract frequent subsequences to discriminate localizations of related proteins [3].

SPAAN (Software for Prediction of Adhesins and Adhesin-like Proteins using Neural Networks) predicts the adhesion probability by using artificial neural networks to use the physiochemical properties (frequencies of amino acids, multiplet and dipeptide as well as charge and hydrophobic composition) of known adhesions and non-adhesion molecules as training data [4].

TMHMM (Transmembrane Helices Hidden Markov Models) (v2.0) predicts the number of transmembrane helices by using hidden Markov models to model various regions of membrane proteins (helix caps, middle of helix regions close to membrane, and globular domains) by incorporating hydrophobicity, charge bias, helix lengths, and grammatical constrains [5].

Protein Identity

GenBank, RefSeq, and similar *Salmonella* protein descriptions were queried for keywords to identify proteins for potential manual curation based upon their protein identity. Plasmid-associated proteins were searched for using “plasmid”, flagellar-associated proteins using “flagellar” or “flagellin”, and lipopolysaccharide-associated using “LPS” or “lipopolysaccharide”.

VaxiJen2

VaxiJen (v2.0) ([http://www.ddg-pharmfac.net/vaxijen/VaxiJen/VaxiJen .html](http://www.ddg-pharmfac.net/vaxijen/VaxiJen/VaxiJen%20.html)) is an alignment-independent method for antigen prediction based upon auto cross covariance transformation of protein sequences into uniform equal length vectors [6]. The amino acid hydrophobicity, molecular size, and polarity represented by z descriptors are used as inputs into the method.

Protein Sequence Conservation Analysis

The BLAST (Basic Local Alignment Search Tool) E-value is the number of alignments that could be found by chance when searching a query against a database of a particular size while considering the length of the query sequence. The smaller the E-value, the better the match.

Phase II – Epitope Identification & Filtering

CTL and HTL Epitope Prediction

NetMHCpan (v.4.1) (<https://services.healthtech.dtu.dk/services/NetMHCpan-4.1/>), which is used to find CTL epitopes, and NetMHCIIpan (v.4.0) (<https://services.healthtech.dtu.dk/services/NetMHCIIpan-4.0/>), which is used to find HTL epitopes, were trained on two datasets: peptide-MHC binding affinity data and eluted ligands (EL)from mass spectrometry (MS) peptidome data [7-9]. The latter dataset was gathered from MHC molecules that were immunopurified from lysed antigen-presenting cells (APCs), then bound peptides were chromatographically eluted, and finally sequenced by MS/MS [9]. The data includes comprehensive signals from antigen presentation including antigen digestion, MHC loading of ligands, and cell surface transport. This additional data also allows for consideration of length preference in epitope predictions. Both the binding affinity and eluted ligand data are used as inputs into neural network training using the NNAlign training approach with insertions and deletions

Immunogenicity and Antigenicity

IEDB (Immune Epitope Database) Class I Immunogenicity (<http://tools.iedb.org/immunogenicity/>) is used to determine which CTL epitopes are considered immunogenic [10]. When comparing immunogenic to non-immunogenic peptide-MHC complexes, as determined by peptide-immunization experiments, the authors of this tool determined that that T-cells prefer certain amino acids (aromatic and large residues). They also determined which part of the peptide (P4-P6 of a 9-mer) was the most important to immunogenicity. These results were put into a simple enrichment model, and they found that immunogenicity is to some extent predictable (AUC [area under the receiver operating characteristic curve]=0.65).

Toxicity and Hydrophobicity

ToxinPred (<https://webs.iiitd.edu.in/raghava/toxinpred/>) is used to determined whether peptides or short proteins are toxic or non-toxic [11]. The creators of ToxinPred compared toxic peptides (<35 amino acids) from several databases (ATDB, Arachno-Server, Conoserver, DBETH, BTXpred, NTXpred, and SwissProt) and non-toxic peptides (< 35 amino acids) from Swiss-Prot and trEMBL databases. They determined that some amino acids were more likely to be found in toxic (cysteine) or non-toxic (proline, aspartic acid, histidine) peptides overall and at the N- and C- terminals. They created a dipeptide composition-based model (AUC =0.98) as well as a hybrid model that also considered motif information (MEME suite/Motif Alignment and Search Tool [MAST] method, AUC =0.99).

ToxinPred also provides information on hydrophobicity, hydropathicity, hydrophilicity, charge, and molecular weight by default. The GRAVY Score is calculated by adding the hydropathy value for each residue, each amino acid has an assigned value, and dividing by the length of the sequence [12].

Dual Purpose Linear B Cell Epitope Prediction and Discovery

BepiPred (v3.0) (<https://services.healthtech.dtu.dk/services/BepiPred-3.0/>) is a sequence-based tool that uses numerical representations from the protein language model ESM-2, a deep learning method, to predict both linear and confirmational B-cell lymphocyte (CBL) epitopes (AUC=0.762) [13].

Phase IV – Assessment of Construct Properties

Physical and Chemical Characteristics

The instability index was created based upon the finding that certain dipeptides were statistically determined to occur more frequently in stable (n=32) versus unstable (n=12) proteins [14]. Calculation of the instability index takes into account the peptide length and the weight value of instability assigned to each of the 400 possible dipeptides based upon this data. The aliphatic index is an indication of the construct’s thermostability and is calculated based upon the relative volume occupied by aliphatic side chains (alanine, valine, isoleucine, and leucine) [15].

Antigenicity, Allergenicity, Solubility, and Toxicity

ANTIGENPro, which is part of the SCRATCH Protein Predictor server (<http://scratch.proteomics.ics.uci.edu/>), (AUC=81) is an alignment-free method that computes and predicts a number of sequence-based features from the query and predicts its antigenicity based upon its model [16, 17]. This SVM-based model was trained on data from known protective antigens and reactivity data obtained by protein microarray analysis for five pathogens

AllergenFP (FP means fingerprint) (<https://ddg-pharmfac.net/AllergenFP/>) predicts allergenicity by summarizing the sequence features of a query sequence into five E-descriptors which are then transformed into a uniform vector via auto-and cross-covariance [18]. This vector is then used to generate a binary descriptor fingerprint. The Tanimoto coefficients from this fingerprint are used to calculate the query sequence’s similarity to other proteins in the AllergenFP algorithm to finally predict its allergenicity. The algorithm was trained on features from 2,437 allergens and non-allergens from various sources.

SolPro, which is part of the SCRATCH Protein Predictor server (<http://scratch.proteomics.ics.uci.edu/>), uses its two-stage SVM architecture to predict the solubility propensity of a query protein that is overexpressed in E. coli with an overall accuracy of 74% at a threshold of 0.5 [17, 19]. Its model was trained on the computed or predicted features from the primary sequence of greater than 17,000 non-redundant proteins from subsets of the PDB, SwissProt, TargetDB, and previous studies datasets

The ToxinPred2 server (<https://webs.iiitd.edu.in/raghava/toxinpred2/>) is used to predict the toxicity of a protein via its sequence [20]. The ToxinPred2 prediction is based upon a hybrid approach combining similarity- (BLAST), motif- (MERCI [Motif Emerging and Classes-Identification]) and ML-based models. The algorithm was based upon a main dataset of 8,233 toxic and non-toxic proteins from UniProt (v.2021_03).

Secondary Structure Prediction

PSIPRED (PSI-blat based secondary structure Prediction) (v. 4.0) predicts the secondary structure of a protein by using the output from position specific iterated (PSI)-BLAST as input into two feed-forward neural networks [21, 22].

Tertiary Structure Prediction and Refinement

Phyre (Protein Homology/analogy Recognition Engine) (v. 2.0) (<http://www.sbg.bio.ic.ac.uk/~phyre2/html/page.cgi?id=index>) has a four-stage process to generate a 3D model [23]. In Stage 1 (gathering homologous sequences) HHBlits is used to generate a multiple-sequence alignment, which is used by PSIPRED to predict its secondary structure and then both these outputs are used to create an HMM. In Stage 2 (fold library scanning) the query’s HMM is then compared to o a database of known structures using HHsearch generating a crude backbone that then goes through loop modeling (Stage 3) where indels are corrected. Finally in Stage 4 (side-chain placement) side chains are added to generate a final model. The intensive mode (Stage 3b) uses heuristics to choose models created after Stage 3 to maximize confidence and query sequence coverage, extracts pairwise Calpha-Calpha distances for input into Poing. Poing treats these inputs as linear inelastic springs into a virtual ribosome then predicts regions not covered by templates *ab initio* based upon Poing’s algorithm which uses a solvent bombardment model, predicted secondary structure springs, and penalization of steric classes to reconstruct the backbone with Pulchra using a clustered final model of 100 predicted models.

GalaxyRefine (v. 1.0) (<https://galaxy.seoklab.org/cgi-bin/submit.cgi?type=REFINE>) is used to refine the an initial protein model [24]. GalaxyRefine conducts multiple cycles of rebuilding the side chains, repacking side chains, and then overall structure relaxation (mild and aggressive) by molecular dynamics simulations to generate 32 models. Five of these models are reported with model 1 representing the lowest energy model generated by mild relaxation and models 2-5 representing the models closest to the four largest models generated by aggressive relaxation. GalaxyRefine reports a number of metrics to compare and contrast the original and 5 new models. GDT-HA (Global Distance Test-High Accuracy) measures the protein backbone similarity between the initial model and subsequent alternative models by averaging the percentage of Cα atoms within multiple arbitrarily chosen distance cutoffs (0.5, 1.0, 2.0, and 4.0 Å) between the structures after superimposition [24, 25]. RMSD (root mean square deviation) is a sequence-dependent alignment that maximizes the number of Cα that are within 4 Å of the initial protein such that values closer to 0 indicated alternative models closer to the initial model [25]. MolProbity is a value calculated based upon the clash score (Clash score in GalaxyRefine, number of all-atom steric overlaps > 0.4 per 1000 atoms), rotamer outlier score (poor rotamers in GalaxyRefine, percentage of evaluable side-chains classified as outlier rotamers) and a Ramachandran outlier score (1-Rama favored from GalaxyRefine, percentage of residues with angles outside favored regions of Ramachandran maps) [26].

Tertiary Structure Refinement

Validation of 3D models can be conducted in multiple ways. The SAVES (v. 6.0) server (<https://saves.mbi.ucla.edu/>) is used to run validation checks using ERRAT, VERIFY 3D, and PROCHECK protein structures. ERRAT evaluates the relative frequencies of noncovalent (non-bonded) contacts between atoms in nine-residue fragments of the input structure while assuming that different atom types are non-randomly distributed within respect to each other in proteins [27, 28]. VERIFY3D measures the compatibility of a 3D model with its sequence via three parameters: the area of a buried residue, the fraction of side-chain are that is covered by polar atoms and the local secondary structure [28, 29]. PROCHECK compares the query’s stereochemical properties to a database of high-resolution proteins structures from the Protein Data Bank and summarizes the results in a PostScript output, a detailed residue-by-residue listing, and a Ramachandran plot of residue φ–ψ torsion angles [28, 30]. ProSA-web (Protein Structure Analysis) (<https://prosa.services.came.sbg.ac.at/prosa.php>) uses knowledge-based potentials of mean force to evaluate model accuracy by evaluating the structure energy with distance-based pair potential and a potential that captures the solvent exposure of protein residues to generate a z-score [31].

Molecular Docking

The ClusPro server (v 2.0) (<https://cluspro.bu.edu/home.php>) performs three computational steps to generate protein-protein docking: rigid body docking with their in-lab fast Fourier transform-based Piper, RMSD based clustering of the 1000 lowest energy structures, and removal of steric clashes by energy minimization to generate up to 30 final models [32-35]. It requires curated pdb files as inputs i.e. any elements besides the main ectodomain removed. The Center or Lowest Energy Weighted Scores are based upon the repulsive and attractive contributions to the van der Waals interaction energy, electrostatic energy, and a pairwise structure-based potential constructed by the ‘decoys as the reference state’ approach [32]. Scores are generated for both the centers of the large clusters of low-energy as well as the lowest-energy within the structures as a whole.

Molecular Dynamics

GROMACS (GROningen MAchine for Chemical Simulations) (<https://www.gromacs.org/>) is used for molecular dynamics simulations to evaluate the interactions of particles for a certain amount of time steps. GROMACS employs domain decomposition to partition the simulation for multi-level parallelism to conduct simulations quickly and efficiently [36]. High performance is achieved using dynamic load balancing between domains performed in all three dimensions in triclinic geometry.

Parameters files (.mdp) and procedure for GROMACS were adapted from previous studies [37-39]. The following commands were used for each of the specified stages in the simulation process: *gmx pdb2gmx* to generate a GROMACS-formatted topology file, *gmx editconf* to create a virtual simulation box, *gmx solvate* to all solvates to the system, *gmx genioin* to add ions to a specific concentration, and *gmx mdrun* to run simulations (i.e. energy minimization, NVT, etc..). In the energy minimization simulation, potential energy of the system was minimized by adjusting the atomic coordinates until the maximum force reached.

Calculations of RMSD, RMSF, and radius of gyration require the selection of a group within the protein(s) inputs to calculate the measure on. RMSD was calculated on the backbone group which is the repeating sequence of atoms that form the protein backbone including the amino group (N), the carbon each amino acid residue’s side chain attaches to (Cα), and the carboxyl group (C). RMSF was calculated solely on the Cα group while the radius of gyration as calculated on the protein as whole. The backbone and Cα groups are more characteristic of conformational changes than the more flexible side-chains [38]. Visualizations for each of the RMSD, RMSF, radius of gyration outputs was created with the GROMACS .xvg outputs by using the readXVG from the Peptides R library to convert them to dataframes.

Other relevant details regarding the molecular dynamic simulation steps used in the present study are found in the main manuscript and details regarding the GROMACS algorithms used at each step are beyond the scope of these supplementary notes [40].

Immune Simulation

C-ImmSim (<https://kraken.iac.rm.cnr.it/C-IMMSIM/>) is used to run an in silico vaccination simulation to characterize the immune response to the vaccine construct [41-44]. C-ImmSim is an agent-based simulation where each agent (cell) is individually simulated using a Position Specific Scoring Matric (PSSM)-based method to predict immune interactions across three anatomical regions: bone marrow, thymus, and tertiary lymphatic organs. MHCI and II epitopes are predicted by using a 9-mer sliding window on the peptide to test every possible epitope [43]. Although the length of Class II epitopes can vary up to 30 and have an average of 16+/- 4.2 residues, the binding core can be reduced to a 9-mer. A 9-mer is considered to be an epitope if its C-ImmSim algorithm score is above an allele-specific threshold, then the 1% strongest binders are extracted. LBL epitopes are predicted using the Parker propensity scale to test a smoothing window size of 7 to test a range of amino acids. An amino acid with a propensity above 0.7 is considered a potential epitopic amino acid and only continuous epitopic amino acids lengths of 4 or more are considered to be an LBL. The contact potential between two amino acid strings, either a BCR and an antigen or a TCR and an MHC-peptide complex, is based on the Miyazawa-Jernigan score.

Phase V Conservation of Construct Epitopes

*Salmonella* serovar was originally determined by PulseNet with SeqSero2 [45, 46]; unnamed serovars were noted as “Needs further review” and “Non” in the original metadata. PulseNet metadata columns (source site, source type, and type details) were queried for keywords associated with specific isolations sources in the following order: egg ("egg", "EGG", "Egg"), turkey ("Turkey", "turkey", "Meleagris gallopavo", "meleagris gallopavo"), chicken ("Chicken", "chicken", "Gallus gallus", "Gallus Gallus", "CHICKEN", "chick", "Chick", "CHICK PADS", "broiler", "Broiler", "Layers", "layers"), cattle/beef ("Cow", "cow", "Cattle", "cattle", "Bos taurus", "beef", "Beef", "bovine", "Bovine", "Calf", "calf"), swine/pork ("Swine", "swine", "Sus scrofa", "porcine", "Porcine", "pork", "Pork", "PORK", "Hogs", "pig", "Pig", "Sow", "sow"), and human ("Human", "human", "HUMAN", "Homo sapiens", "homo sapiens"). The Outbreak Source column was queried for the above keywords as well as ones for poultry (“poultry” and “Poultry”).

Bibliography

1.	Ong E, Wang H, Wong MU, Seetharaman M, Valdez N, He Y. Vaxign-ML: supervised machine learning reverse vaccinology model for improved prediction of bacterial protective antigens. Bioinformatics. 2020;36(10):3185-91.

2.	Yang B, Sayers S, Xiang Z, He Y. Protegen: a web-based protective antigen database and analysis system. Nucleic acids research. 2011;39(suppl_1):D1073-D8.

3.	Yu NY, Wagner JR, Laird MR, Melli G, Rey S, Lo R, et al. PSORTb 3.0: improved protein subcellular localization prediction with refined localization subcategories and predictive capabilities for all prokaryotes. Bioinformatics. 2010;26(13):1608-15.

4.	Sachdeva G, Kumar K, Jain P, Ramachandran S. SPAAN: a software program for prediction of adhesins and adhesin-like proteins using neural networks. Bioinformatics. 2005;21(4):483-91.

5.	Krogh A, Larsson B, Von Heijne G, Sonnhammer EL. Predicting transmembrane protein topology with a hidden Markov model: application to complete genomes. Journal of molecular biology. 2001;305(3):567-80.

6.	Doytchinova IA, Flower DR. VaxiJen: a server for prediction of protective antigens, tumour antigens and subunit vaccines. BMC bioinformatics. 2007;8(1):1-7.

7.	Reynisson B, Alvarez B, Paul S, Peters B, Nielsen M. NetMHCpan-4.1 and NetMHCIIpan-4.0: improved predictions of MHC antigen presentation by concurrent motif deconvolution and integration of MS MHC eluted ligand data. Nucleic acids research. 2020;48(W1):W449-W54.

8.	Jurtz V, Paul S, Andreatta M, Marcatili P, Peters B, Nielsen M. NetMHCpan-4.0: improved peptide–MHC class I interaction predictions integrating eluted ligand and peptide binding affinity data. The Journal of Immunology. 2017;199(9):3360-8.

9.	Reynisson B, Barra C, Kaabinejadian S, Hildebrand WH, Peters B, Nielsen M. Improved prediction of MHC II antigen presentation through integration and motif deconvolution of mass spectrometry MHC eluted ligand data. Journal of proteome research. 2020;19(6):2304-15.

10.	Calis JJ, Maybeno M, Greenbaum JA, Weiskopf D, De Silva AD, Sette A, et al. Properties of MHC class I presented peptides that enhance immunogenicity. PLoS computational biology. 2013;9(10):e1003266.

11.	Gupta S, Kapoor P, Chaudhary K, Gautam A, Kumar R, Consortium OSDD, et al. In silico approach for predicting toxicity of peptides and proteins. PloS one. 2013;8(9):e73957.

12.	Kyte J, Doolittle RF. A simple method for displaying the hydropathic character of a protein. Journal of molecular biology. 1982;157(1):105-32.

13.	Clifford JN, Høie MH, Deleuran S, Peters B, Nielsen M, Marcatili P. BepiPred‐3.0: Improved B‐cell epitope prediction using protein language models. Protein Science. 2022;31(12):e4497.

14.	Guruprasad K, Reddy BB, Pandit MW. Correlation between stability of a protein and its dipeptide composition: a novel approach for predicting in vivo stability of a protein from its primary sequence. Protein Engineering, Design and Selection. 1990;4(2):155-61.

15.	Ikai A. Thermostability and aliphatic index of globular proteins. The Journal of Biochemistry. 1980;88(6):1895-8.

16.	Magnan CN, Zeller M, Kayala MA, Vigil A, Randall A, Felgner PL, et al. High-throughput prediction of protein antigenicity using protein microarray data. Bioinformatics. 2010;26(23):2936-43.

17.	Cheng J, Randall AZ, Sweredoski MJ, Baldi P. SCRATCH: a protein structure and structural feature prediction server. Nucleic Acids Research. 2005;33(suppl_2):W72-W6.

18.	Dimitrov I, Naneva L, Doytchinova I, Bangov I. AllergenFP: allergenicity prediction by descriptor fingerprints. Bioinformatics. 2014;30(6):846-51.

19.	Magnan CN, Randall A, Baldi P. SOLpro: accurate sequence-based prediction of protein solubility. Bioinformatics. 2009;25(17):2200-7.

20.	Sharma N, Naorem LD, Jain S, Raghava GP. ToxinPred2: An improved method for predicting toxicity of proteins. Briefings in Bioinformatics. 2022;23(5):bbac174.

21.	Buchan DW, Jones DT. The PSIPRED protein analysis workbench: 20 years on. Nucleic acids research. 2019;47(W1):W402-W7.

22.	Jones DT. Protein secondary structure prediction based on position-specific scoring matrices. Journal of molecular biology. 1999;292(2):195-202.

23.	Kelley LA, Mezulis S, Yates CM, Wass MN, Sternberg MJE. The Phyre2 web portal for protein modeling, prediction and analysis. Nature Protocols. 2015;10(6):845-58.

24.	Heo L, Park H, Seok C. GalaxyRefine: Protein structure refinement driven by side-chain repacking. Nucleic acids research. 2013;41(W1):W384-W8.

25.	Modi V, Dunbrack Jr RL. Assessment of refinement of template‐based models in CASP11. Proteins: Structure, Function, and Bioinformatics. 2016;84:260-81.

26.	Chen VB, Arendall WB, 3rd, Headd JJ, Keedy DA, Immormino RM, Kapral GJ, et al. MolProbity: all-atom structure validation for macromolecular crystallography. Acta Crystallogr D Biol Crystallogr. 2010;66(Pt 1):12-21.

27.	Colovos C, Yeates TO. Verification of protein structures: patterns of nonbonded atomic interactions. Protein science. 1993;2(9):1511-9.

28.	Rossmann MG, Arnold E. International Tables for Crystallography Volume F: Crystallography of biological macromolecules: Wiley Online Library; 2001.

29.	Eisenberg D, Lüthy R, Bowie JU. VERIFY3D: assessment of protein models with three-dimensional profiles. Methods in enzymology. 277: Elsevier; 1997. p. 396-404.

30.	Laskowski RA, MacArthur MW, Moss DS, Thornton JM. PROCHECK: a program to check the stereochemical quality of protein structures. Journal of applied crystallography. 1993;26(2):283-91.

31.	Wiederstein M, Sippl MJ. ProSA-web: interactive web service for the recognition of errors in three-dimensional structures of proteins. Nucleic Acids Research. 2007;35(suppl_2):W407-W10.

32.	Kozakov D, Hall DR, Xia B, Porter KA, Padhorny D, Yueh C, et al. The ClusPro web server for protein–protein docking. Nature Protocols. 2017;12(2):255-78.

33.	Desta IT, Porter KA, Xia B, Kozakov D, Vajda S. Performance and its limits in rigid body protein-protein docking. Structure. 2020;28(9):1071-81. e3.

34.	Kozakov D, Beglov D, Bohnuud T, Mottarella SE, Xia B, Hall DR, et al. How good is automated protein docking? Proteins: Structure, Function, and Bioinformatics. 2013;81(12):2159-66.

35.	Vajda S, Yueh C, Beglov D, Bohnuud T, Mottarella SE, Xia B, et al. New additions to the C lus P ro server motivated by CAPRI. Proteins: Structure, Function, and Bioinformatics. 2017;85(3):435-44.

36.	Abraham MJ, Murtola T, Schulz R, Páll S, Smith JC, Hess B, et al. GROMACS: High performance molecular simulations through multi-level parallelism from laptops to supercomputers. SoftwareX. 2015;1-2:19-25.

37.	Abraham M, , Alekseenko A, , Bergh C, , et al. GROMACS 2023.3 Manual 2023 [Available from: <https://zenodo.org/records/10017699>.

38.	CompChems. GROMACS Tutorial: Molecular Dynamics simulation of a protein in water environment 2023 [Available from: <https://www.compchems.com/gromacs-tutorial-molecular-dynamics-simulation-of-a-protein-in-water-environment/>.

39.	Dimitrov D. GROMACS Tutorial. A Guide to Molecular Dynamics Using GROMACS [Available from: <https://static.igem.org/mediawiki/2021/d/d2/T--KCL_UK--UPDATEDGROMACSGUIDE.pdf>.

40.	Pronk S, Páll S, Schulz R, Larsson P, Bjelkmar P, Apostolov R, et al. GROMACS 4.5: a high-throughput and highly parallel open source molecular simulation toolkit. Bioinformatics. 2013;29(7):845-54.

41.	Castiglione F, Bernaschi M. C-ImmSim: playing with the immune response. – Katholieke Universiteit Leuven. 2004.

42.	Ragone C, Manolio C, Cavalluzzo B, Mauriello A, Tornesello ML, Buonaguro FM, et al. Identification and validation of viral antigens sharing sequence and structural homology with tumor-associated antigens (TAAs). Journal for ImmunoTherapy of Cancer. 2021;9(5):e002694.

43.	Rapin N, Lund O, Bernaschi M, Castiglione F. Computational Immunology Meets Bioinformatics: The Use of Prediction Tools for Molecular Binding in the Simulation of the Immune System. PLoS ONE. 2010;5(4):e9862.

44.	Stolfi P, Castiglione F, Mastrostefano E, Di Biase I, Di Biase S, Palmieri G, et al. In-silico evaluation of adenoviral COVID-19 vaccination protocols: Assessment of immunological memory up to 6 months after the third dose. Frontiers in immunology. 2022;13:998262.

45.	Swaminathan B, Barrett TJ, Hunter SB, Tauxe RV. PulseNet: the molecular subtyping network for foodborne bacterial disease surveillance, United States. Emerg Infect Dis. 2001;7(3):382-9.

46.	Tolar B, Joseph LA, Schroeder MN, Stroika S, Ribot EM, Hise KB, et al. An Overview of PulseNet USA Databases. Foodborne Pathog Dis. 2019;16(7):457-62.
