# Supplementary material for: In silico design and evaluation of a candidate multiepitope Salmonella vaccine construct targeting broad-spectrum protection in poultry
Source: BMC Genomics. 2026 Jun 25;27:758. doi: 10.1186/s12864-026-13056-4 (PMC13584361; doi:10.1186/s12864-026-13056-4)
Supplement: Supplementary file 2 [file 12864_2026_13056_MOESM4_ESM.docx]

Supplementary Figure 1: Secondary structure prediction of the multiepitope vaccine construct by PSIPRED server. Graphical representation of the predicted secondary structure type for each multiepitope vaccine construct residue indicated by box fill and edge color.

Supplementary Figure 2: Molecular dynamics simulation of multiepitope vaccine construct docked to the TLR1/2 heterodimer. GROMACS pre-production run results are summarized as the: (A) potential energy of system over time, (B) RMSD (root mean square deviation) of NVT (constant number of particles, volume, and temperature) simulation over time, (C) temperature of NVT simulation over time, (D) RMSD of NPT (constant number of particles, pressure, and temperature) simulation over time, and (E) pressure of NPT simulation over time.

Supplementary Figure 3: Immune simulation of MHCI HLA-B*40:06 and MHCII DRBI*13:10 alleles with the multiepitope construct. C-ImmSim server simulation was run with injections of the multiepitope vaccine construct occurring at Days 0 and 21. (A) Antigens and immunoglobin levels, (B) production of cytokine and interleukins with Simpson index D, (C) macrophage population per state, (D) dendritic cell population per state , (E) B lymphocyte population per state, (F) B lymphocyte population, (G) cytotoxic T lymphocyte (TC) population, (H) helper T lymphocyte (TH) population per state, and (I) helper T lymphocyte (TH) population.

Supplementary Figure 4: Immune simulation of MHCI HLA-B*41:03 and MHCII DRBI*13:66 alleles with the multiepitope construct. C-ImmSim server simulation was run with injections of the multiepitope vaccine construct occurring at Days 0 and 21. (A) Antigens and immunoglobin levels, (B) production of cytokine and interleukins with Simpson index D, (C) macrophage population per state, (D) dendritic cell population per state , (E) B lymphocyte population per state, (F) B lymphocyte population, (G) cytotoxic T lymphocyte (TC) population, (H) helper T lymphocyte (TH) population per state, and (I) helper T lymphocyte (TH) population.

Supplementary Figure 5: Alignment of multiepitope vaccine construct (MEVC) epitopes to PulseNet outbreak-associated proteomes by isolation source. The color gradient (100% = dark blue, 50% = purple, 0% = dark red) in the heatmap illustrates the percentage of PulseNet outbreak-associated proteomes with complete homology (100% sequence identity and coverage) to each MEVC epitope (n = 28). Percentages are provided per specified isolation source (n = 6) as well as an “Other” category that includes isolation sources that were not the focus of this study (n = 1,021 isolates). The specified isolation sources included: human- (n = 31,012), chicken- (n = 726), turkey- (n = 377), cattle/beef- (n = 270), swine/pork- (n = 260), and egg-associated (n = 6) sources. Colors on the left-hand side of the heatmap indicate the epitope type.
